# Meta-GWAS Accuracy and Power (MetaGAP) Calculator Shows that Hiding Heritability Is Partially Due to Imperfect Genetic Correlations across Studies

**DOI:** 10.1371/journal.pgen.1006495

**Published:** 2017-01-17

**Authors:** Ronald de Vlaming, Aysu Okbay, Cornelius A. Rietveld, Magnus Johannesson, Patrik K. E. Magnusson, André G. Uitterlinden, Frank J. A. van Rooij, Albert Hofman, Patrick J. F. Groenen, A. Roy Thurik, Philipp D. Koellinger

**Affiliations:** 1 Erasmus University Rotterdam Institute for Behavior and Biology, Erasmus School of Economics, Rotterdam, the Netherlands; 2 Department of Applied Economics, Erasmus School of Economics, Rotterdam, the Netherlands; 3 Department of Complex Trait Genetics, Center for Neurogenomics and Cognitive Research, Amsterdam, the Netherlands; 4 Department of Economics, Stockholm School of Economics, Stockholm, Sweden; 5 Department of Medical Epidemiology and Biostatistics, Karolinska Institutet, Stockholm, Sweden; 6 Department of Epidemiology, Erasmus University Medical Center Rotterdam, Rotterdam, the Netherlands; 7 Department of Epidemiology, Harvard T.H. Chan School of Public Health, Boston, Massachusetts, United States of America; 8 Econometric Institute, Erasmus School of Economics, Rotterdam, the Netherlands; 9 Montpellier Business School, Montpellier, France; University of Oxford, UNITED KINGDOM

## Abstract

Large-scale genome-wide association results are typically obtained from a fixed-effects meta-analysis of GWAS summary statistics from multiple studies spanning different regions and/or time periods. This approach averages the estimated effects of genetic variants across studies. In case genetic effects are heterogeneous across studies, the statistical power of a GWAS and the predictive accuracy of polygenic scores are attenuated, contributing to the so-called ‘missing heritability’. Here, we describe the online Meta-GWAS Accuracy and Power (MetaGAP) calculator (available at www.devlaming.eu) which quantifies this attenuation based on a novel multi-study framework. By means of simulation studies, we show that under a wide range of genetic architectures, the statistical power and predictive accuracy provided by this calculator are accurate. We compare the predictions from the MetaGAP calculator with actual results obtained in the GWAS literature. Specifically, we use genomic-relatedness-matrix restricted maximum likelihood to estimate the SNP heritability and cross-study genetic correlation of height, BMI, years of education, and self-rated health in three large samples. These estimates are used as input parameters for the MetaGAP calculator. Results from the calculator suggest that cross-study heterogeneity has led to attenuation of statistical power and predictive accuracy in recent large-scale GWAS efforts on these traits (e.g., for years of education, we estimate a relative loss of 51–62% in the number of genome-wide significant loci and a relative loss in polygenic score *R*^2^ of 36–38%). Hence, cross-study heterogeneity contributes to the missing heritability.

## Author Summary

Large-scale genome-wide association studies are uncovering the genetic architecture of traits which are affected by many genetic variants. In such efforts, one typically meta-analyzes association results from multiple studies spanning different regions and/or time periods. Results from such efforts do not yet capture a large share of the heritability. The origins of this so-called ‘missing heritability’ have been strongly debated. One factor exacerbating the missing heritability is heterogeneity in the effects of genetic variants across studies. The effect of this type of heterogeneity on statistical power to detect associated genetic variants and the accuracy of polygenic predictions is poorly understood. In the current study, we derive the precise effects of heterogeneity in genetic effects across studies on both the statistical power to detect associated genetic variants as well as the accuracy of polygenic predictions. We present an online calculator, available at www.devlaming.eu, which accounts for these effects. By means of this calculator, we show that imperfect genetic correlations between studies substantially decrease statistical power and predictive accuracy and, thereby, contribute to the missing heritability. The MetaGAP calculator helps researchers to gauge how sensitive their results will be to heterogeneity in genetic effects across studies. If strong heterogeneity is expected, random-effects meta-analysis methods should be used instead of fixed-effects methods.

## Introduction

Large-scale GWAS efforts are rapidly elucidating the genetic architecture of polygenic traits, including anthropometrics [1, 2] and diseases [3–5], as well as behavioral and psychological outcomes [6–8]. These efforts have led to new biological insights, therapeutic targets, and polygenic scores (PGS), and help to understand the complex interplay between genes and environments in shaping individual outcomes [7, 9, 10]. However, GWAS results do not yet account for a large part of the estimated heritability [1, 2, 7, 8]. This dissonance, which is referred to as the ‘missing heritability’, has received broad attention [11–17].

Differences across strata (e.g., studies and populations), in genetic effects, phenotype measurement, and phenotype accuracy, lead to loss of signal [18–20]. Hence, such forms of heterogeneity attenuate the statistical power of a GWAS [17, 18, 21, 22] and the predictive accuracy of a PGS in a hold-out sample [23], and, thereby, contribute to the missing heritability. Since large-scale GWAS results are typically obtained from a meta-analysis of GWAS results from many different studies, we focus on the attenuation resulting from heterogeneity at the level of studies included in such a meta-analysis. Given the importance of discovering trait-affecting variants and obtaining accurate polygenic predictions, it is vital to understand to which extent cross-study heterogeneity attenuates the statistical power and predictive accuracy of GWAS efforts. By considering cross-study differences in genetic effects and heritability, we can quantify this attenuation.

Despite empirical evidence of transethnic genetic heterogeneity in diseases [24] and the fact that cross-study heterogeneity has been found to decrease the chances of a study to yield meaningful results [22, 25], a theoretical multi-study framework that quantifies the effect of cross-study heterogeneity on statistical power and predictive accuracy is still absent. We bridge this gap by developing a Meta-GWAS Accuracy and Power (MetaGAP) calculator (available at www.devlaming.eu) that accounts for the cross-study genetic correlation (CGR). This calculator infers the statistical power to detect associated SNPs and the predictive accuracy of the PGS in a meta-analysis of GWAS results from genetically and phenotypically heterogeneous studies, and quantifies the loss in power and predictive accuracy incurred by this cross-study heterogeneity. Using simulations, we show that the MetaGAP calculator is accurate under a wide range of genetic architectures, even when the assumptions of the calculator are violated.

Although meta-analysis methods accounting for heterogeneity exist [26–31], large-scale GWAS results are typically still obtained from fixed-effects meta-analysis methods [32, 33] such as implemented in METAL [34]. Therefore, the MetaGAP calculator assumes the use of a fixed-effects meta-analysis method. Thus, the calculator will help researchers to assess the merits of an intended fixed-effects meta-analysis of GWAS results and to gauge whether it is more appropriate to apply a meta-analysis method that accounts for heterogeneity.

In an empirical application, we use genomic-relatedness-matrix restricted maximum likelihood (GREML) to estimate the SNP-based heritability ($h_{\text{SNP}}^{2}$) and CGR of several polygenic traits across three distinct studies: the Rotterdam Study (RS), the Swedish Twin Registry (STR), and the Health and Retirement Study (HRS). For self-rated health, years of education, BMI, and height, we obtain point-estimates of CGR between 0.47 and 0.97. Based on these estimates of $h_{\text{SNP}}^{2}$ and CGR, we use the MetaGAP calculator to quantify the expected number of hits and predictive accuracy of the PGS in recent GWAS efforts for these traits. Our theoretical predictions align with empirical observations.

For height, under an estimated CGR of 0.97, the expected relative loss in the number of genome-wide significant hits is 8–9%, whereas, for years of education, under an estimated CGR of 0.78, we expect a relative loss of 51–62% in the number of hits. Moreover, we find that the relative loss in PGS *R*^2^ is expected to be 6–7% for height and 36–38% for years of education. Hence, our findings show that cross-study heterogeneity attenuates the statistical power and PGS accuracy considerably, thus, contributing substantially to the missing heritability, and, more specifically, to the ‘hiding heritability’ [15–17]—defined as the difference between the SNP-based heritability estimate [35] and the proportion of phenotypic variation explained by genetic variants that reach genome-wide significance in a GWAS.

## Materials and Methods

### Definitions and assumptions

The MetaGAP calculator is based on theoretical expressions for statistical power and PGS accuracy, derived in S1 Derivations and S2 Derivations. In these expressions, within-study estimates of SNP heritability (e.g., inferred using GCTA [36]) are required input parameters. Estimates of CGR (e.g., inferred as genetic correlations across studies using pairwise bivariate methods as implemented in GCTA [37] and LD-score regression [38, 39], or as genetic-impact correlation from summary statistics [24]) also play a central role in those expressions. As we show in S1 Note, such estimates of CGR are affected by the cross-study overlap in trait-affecting loci as well as the cross-study correlation in the effects of these overlapping loci. In our derivations of statistical power and predictive accuracy, we assume, however, that the set of trait-affecting loci is the same across all studies and that CGRs are, consequently, shaped solely by cross-study correlations in the effects. Using simulation studies, discussed in S1 Simulations, we assess how violations of this assumption affect our results.

In addition, genetic correlations as inferred using GCTA [37] or LD-score regression [39] effectively estimate the cross-trait and/or cross-study correlation in the effects of standardized SNPs. This correlation has been referred to as the genetic-impact correlation [24]. The scale of rare variants is inflated most by standardization (i.e., genotypes are scaled by $1/\sqrt{2f(1-f)}$, where *f* denotes the allele frequency of the SNP of interest). Therefore, the scale of the effects of these variants is decreased most by standardization of SNPs (i.e., when standardizing a SNP, the effect is scaled by $\sqrt{2f(1-f)}$). Hence, the genetic-impact correlation emphasizes the contribution of common variants [24]. If rare alleles tend to have larger effects than common alleles, as assumed in GCTA [36] and LD-score regression [38], these two opposing forces may cancel each other out; the effects of rare alleles are then bigger, but also scaled downwards more strongly by considering standardized SNPs. Alternatively, one can also consider the correlation in the effect of non-standardized SNPs, referred to as the genetic-effect correlation [24]. This genetic-effect correlation gives rare and common variants equal weight in theory. However, in case rare alleles have larger effects than common alleles, this genetic-effect correlation, in practice, gives a disproportional weight to rare variants.

A clear definition of genetic correlation can be further complicated by the presence of allele frequency differences across samples. Whereas GCTA assumes fixed allele frequencies across the samples included in the analysis [36], there also exist methods which allow for differences in allele frequencies. Ideally, estimates of cross-study genetic-impact correlation accounting for allele frequency differences [24] should be used in the MetaGAP calculator as input for CGR. However, provided the genetic drift is small, whether to account for allele frequency differences across samples or not, will—in all likelihood—hardly affect the CGR estimates. Therefore, under little genetic drift, estimates of CGR obtained by methods ignoring cross-study differences in allele frequencies (e.g., bivariate GREML [37]), suffice as input for the MetaGAP calculator.

In line with other work, we define the effective number of SNPs, *S*, as the number of haplotype blocks (i.e., independent chromosome segments) [40], where variation in each block is tagged by precisely one genotyped SNP. By genotyped SNPs we also mean imputed SNPs. Hence, in our framework, there are *S* SNPs contributing to the polygenic score. Due to linkage disequilibrium (LD) this number is likely to be substantially lower than the total number of SNPs in the genome [41], and is inferred to lie between as little as 60,000 [15] and as much as 5 million [41].

In terms of trait-affecting variants, we consider a subset of *M* SNPs from the set of *S* SNPs. Each SNP in this subset tags variation in a segment that bears a causal influence on the phenotype. We refer to *M* as the associated number of SNPs. We assume that the *M* associated SNPs jointly capture the full SNP-based heritability for the trait of interest and, moreover, that each associated SNP has the same theoretical *R*^2^ with respect to the phenotype. In the simulation studies, we also assess the impact of violations of this ‘equal-*R*^2^’ assumption.

By considering only independent genotyped SNPs that are assumed to fully tag the causal variants, we can ignore LD among genotyped variants and between the causal variant and the genotyped variants. Thereby, we can greatly reduce the theoretical and numerical complexity of the MetaGAP calculator. However, a genotyped tag SNP does not necessarily capture the full variation of the causal variant present in that independent segment. Nevertheless, the inputs for SNP heritability used in the MetaGAP calculator are within-study GREML estimates of heritability, based on the available SNPs. Therefore, if these genotyped SNPs are in imperfect LD with the causal variants, this will lead to a downward bias in the SNP-based heritability estimates [42]. Hence, the imperfect tagging of the causal variants is likely to be absorbed by a downward bias in the SNP-based heritability estimates.

### Power of a GWAS meta-analysis under heterogeneity

The theoretical distribution of the *Z* statistic, resulting from a meta-analysis of GWAS results under imperfect CGRs, can be found in S1 Derivations. These expressions allow for differences in sample size, $h_{\text{SNP}}^{2}$, and CGR across (pairs of) studies. For intuition, we here present the specific case of a meta-analysis of results from two studies with CGR *ρ*_**G**_, with equal SNP-based heritability $h_{\text{SNP}}^{2}$, and equal sample sizes (i.e., *N* in Study 1 and *N* in Study 2). Under this scenario, we find that under high polygenicity, the *Z* statistic of an associated SNP *k* is normally distributed with mean zero and the following variance:

$$\text{Var}\left(Z_{k}\right)=E\left[Z_{k}^{2}\right]\approx 1+\frac{h_{\text{SNP}}^{2}}{M}N\left(1+\rho_{G}\right).$$ (1)

We incorporate cross-study genetic heterogeneity by assuming that the data-generating process follows a random-effects model, where cross-study correlations in SNP effects shape the inferred CGRs. When one has random effects, under the null hypothesis a SNP effect follows a degenerate distribution with all probability mass at zero, whereas under the alternative hypothesis a SNP effect follows a distribution with mean zero and a finite non-zero variance. Bearing in mind that we can write a meta-analysis *Z* statistic as a weighted average of true effects across studies and noise terms, the null hypothesis leads to a *Z* statistic with a mean equal to zero and a variance equal to one, whereas the alternative hypothesis does not lead to a non-zero mean in the *Z* statistic, but rather to excess variation (i.e., a variance larger than one).

The larger the variance in the *Z* statistic, the higher the probability of rejecting the null. The ratio of $h_{\text{SNP}}^{2}$ and *M* can be regarded as the theoretical *R*^2^ of each associated SNP with respect to the phenotype. Eq 1 reveals that (i) when sample size increases, power increases, (ii) when $h_{\text{SNP}}^{2}$ increases, the *R*^2^ per associated SNP increases and therefore power increases, (iii) when the number of associated SNPs increases, the *R*^2^ per associated SNP decreases and therefore power decreases, (iv) when the CGR is zero the power of the meta-analysis is identical to the power obtained in each of the two studies when analyzed separately, yielding no strict advantage to meta-analyzing, and (v) when the CGR is positive one, the additional variance in the *Z* statistic—compared to the variance under the null—is twice the additional variance one would have when analyzing the studies separately, yielding a strong advantage to meta-analyzing.

Notably, our expression for $E\;[Z_{k}^{2}]$ bears a great resemblance to expressions for the expected value of the squared *Z* statistic when accounting for LD, population stratification, and polygenicity [38, 43, 44]. Consider the scenario where the CGR between two samples of equal size is positive one. Based of Eq 1, we then have that $E\;[Z_{k}^{2}]\approx 1+\frac{h_{\text{SNP}}^{2}}{M}N_{T}$ for a trait-affecting haplotype block, where *N_T_* = 2*N* denotes the total sample size. This expression is equivalent to the expected squared *Z* statistic from the linear regression analysis for a trait-affecting variant reported in Section 4.2 of the Supplementary Note to [44] as well as the first equation in [38] when assuming that confounding biases and LD are absent.

In order to compute statistical power in a multi-study setting, we first use the generic expression for the variance of the GWAS *Z* statistic derived in S1 Derivations to characterize the distribution of the *Z* statistic under the alternative hypothesis. Given a genome-wide significance threshold (denoted by *α*; usually *α* = 5 ⋅ 10^−8^), we use the normal cumulative distribution function under the alternative hypothesis to quantify the probability of attaining genome-wide significance for an associated SNP. This probability we refer to as the ‘power per associated SNP’ (denoted here by *β*). Given that we use SNPs tagging independent haplotype blocks, we can calculate the probability of rejecting the null for at least one SNP and the expected number of hits, true positives, false positives, false negatives, and positive negatives, as functions of *α*, *β*, the number of truly associated SNPs (denoted by *M*), and the number of non-associated SNPs (denoted by *S* − *M*). Letting ‘#’ denote the number of elements in a set, we have that

$$\begin{array}{ll} \mathbb{P}\;\left[\#\text{ true positives }\geq \;1\right] & =1-{\left(1-\beta \right)}^{M},\\ \mathbb{P}\;\left[\#\text{ hits }\geq \;1\right] & =1-\left[{\left(1-\beta \right)}^{M}{\left(1-\alpha \right)}^{S-M}\right],\\ E\;\left[\#\text{ hits}\right] & =\beta M+\alpha \left(S-M\right),\\ E\;\left[\#\text{ true positives}\right] & =\beta M,\\ E\;\left[\#\text{ false positives}\right] & =\alpha (S-M),\\ E\;\left[\#\text{ false negatives}\right] & =\left(1-\beta \right)M,\text{ and}\\ E\;\left[\#\text{ true negatives}\right] & =\left(1-\alpha \right)\left(S-M\right). \end{array}$$

### *R*^2^ of a polygenic score under heterogeneity

In S2 Derivations we derive a generic expression for the theoretical *R*^2^ of a PGS in a hold-out sample, with SNP weights based on a meta-analysis of GWAS results under imperfect CGRs. We consider a PGS that includes all the SNPs that tag independent haplotype blocks (i.e., there is no SNP selection).

For intuition, we here present an approximation for prediction in a hold-out sample, with SNP weights based on a GWAS in a single discovery study with sample size *N*, where both studies have SNP heritability $h_{\text{SNP}}^{2}$, and with CGR *ρ*_**G**_, between the studies. Under high polygenicity, the *R*^2^ of the PGS in the hold-out sample is then given by the following expression:

$$R^{2}\approx h_{\text{SNP}}^{2}\rho_{G}^{2}\frac{h_{\text{SNP}}^{2}}{\frac{S}{N}+h_{\text{SNP}}^{2}}.$$ (2)

In case the CGR is one, and we consider the *R*^2^ between the PGS and the genetic value (i.e., the genetic component of the phenotype) instead of the phenotype itself, the first two terms in Eq 2 disappear, yielding an expression equivalent to the first equation in [40]. Assuming a CGR of one and that all SNPs are associated, Eq 2 is equivalent to the expression in [23] for the *R*^2^ between the PGS and the phenotype in the hold-out sample.

From Eq 2, we deduce that (i) as the effective number of SNPs S increases, the *R*^2^ of the PGS deteriorates (since every SNP-effect estimate contains noise, owing to imperfect inferences in finite samples), (ii) given the effective number of SNPs, under a polygenic architecture, the precise fraction of effective SNPs that is associated does not affect the *R*^2^, (iii) *R*^2^ is quadratically proportional to *ρ*_**G**_, implying a strong sensitivity to CGR, and (iv) as the sample size of the discovery study grows, the upper limit of the *R*^2^ is given by $h_{\text{SNP}}^{2}\rho_{G}^{2}$, implying that the full SNP heritability in the hold-out sample cannot be entirely captured as long as CGR is imperfect.

### Online power and *R*^2^ calculator

An online version of the MetaGAP calculator can be found at www.devlaming.eu. This calculator computes the theoretical power per trait-affecting haplotype block, the power to detect at least one of these blocks, and the expected number of (a) independent hits, (b) true positives, (c) false positives, (d) false negatives, and (e) true negatives, for a meta-analysis of GWAS results from *C* studies. In addition, it provides the expected *R*^2^ of a PGS for a hold-out sample, including all GWAS SNPs, with SNP weights based on the meta-analysis of the GWAS results from *C* studies. Calculations are based on the generic expressions for GWAS power derived in S1 Derivations and PGS *R*^2^ derived in S2 Derivations.

The calculator assumes a quantitative trait. Users need to specify either the average sample size per study or the sample size of each study separately. In addition, users need to specify either the average within-study SNP heritability or the SNP heritability per study. The SNP heritability in the hold-out sample also needs to be provided. Users are required to enter the effective number of causal SNPs and the effective number of SNPs in total. The calculator assumes a fixed CGR between all pairs of studies included in the meta-analysis and a fixed CGR between the hold-out sample and each study in the meta-analysis. Hence, one needs to specify two CGR values: one for the CGR within the set of meta-analysis studies and one to specify the genetic overlap between the hold-out sample and the meta-analysis studies.

Finally, a more general version of the MetaGAP calculator is provided in the form of MATLAB code (www.mathworks.com), also available at www.devlaming.eu. This code can be used in case one desires to specify a more versatile genetic-correlation matrix, where the CGR can differ between all pairs of studies. Therefore, this implementation requires the user to specify a full (*C*+1)-by-(*C*+1) correlation matrix. Calculations in this code are also fully in line with the generic expressions in S1 Derivations and S2 Derivations.

### Assessing validity of theoretical power and *R*^2^

We simulate data for a wide range of genetic architectures in order to assess the validity of our theoretical framework. As we show in S1 Simulations, the theoretical expressions we derive for power and *R*^2^ are accurate, even for data generating processes substantially different from the process we assume in our derivations. Our strongest assumptions are that all truly associated SNPs have equal *R*^2^ with respect to the phenotype, regardless of allele frequency, and that genome-wide CGRs are shaped solely by the cross-study correlations in the effects of causal SNPs. When we simulate data where the former assumption fails and where—in addition—allele frequencies are non-uniformly distributed and different across studies, the root-mean-square prediction error of statistical power lies below 3% and that of PGS *R*^2^ below 2%. Moreover, when we simulate data where the CGR is shaped by both non-overlapping causal loci across studies and the correlation of the effects of the overlapping loci, the RMSE is less than 2% for both statistical power and PGS *R*^2^.

### Estimating SNP heritability and CGR

Using 1000-Genomes imputed data from the RS, STR, and HRS, we estimate SNP-based heritability and CGR respectively by means of univariate and bivariate GREML [36, 37] as implemented in GCTA [36]. In our analyses we consider the subset of HapMap3 SNPs available in the 1000-Genomes imputed data. In S1 Data we report details on the genotype and phenotype data, as well as our quality control (QC) procedure. After QC we have a dataset, consisting of ≈ 1 million SNPs and ≈ 20,000 individuals, from which we infer $h_{\text{SNP}}^{2}$ and CGR. In S1 Estimation we provide details on the specifications of the models used for GREML estimation.

### Ethics statement

Written informed consent was provided by all participants and the research project was approved by the Ethics Committee of Erasmus Medical Center (MEC 02.1015), the Ethics Committee of Stockholm (2007-644-31, 2011-463-32, 2012/270-31/2), the ERIM Institutional Review Board (2014-04), and dbGaP (#3544, #5752, #5082, #5285).

## Results

### Determinants of GWAS power and PGS *R*^2^

Using the MetaGAP calculator, we assessed the theoretical power of a meta-analysis of GWAS results from genetically heterogeneous studies and the theoretical *R*^2^ of the resulting PGS in a hold-out sample, for various numbers of studies and sample sizes, and different values of CGR and $h_{\text{SNP}}^{2}$.

#### Sample size and CGR

**Fig 1:**
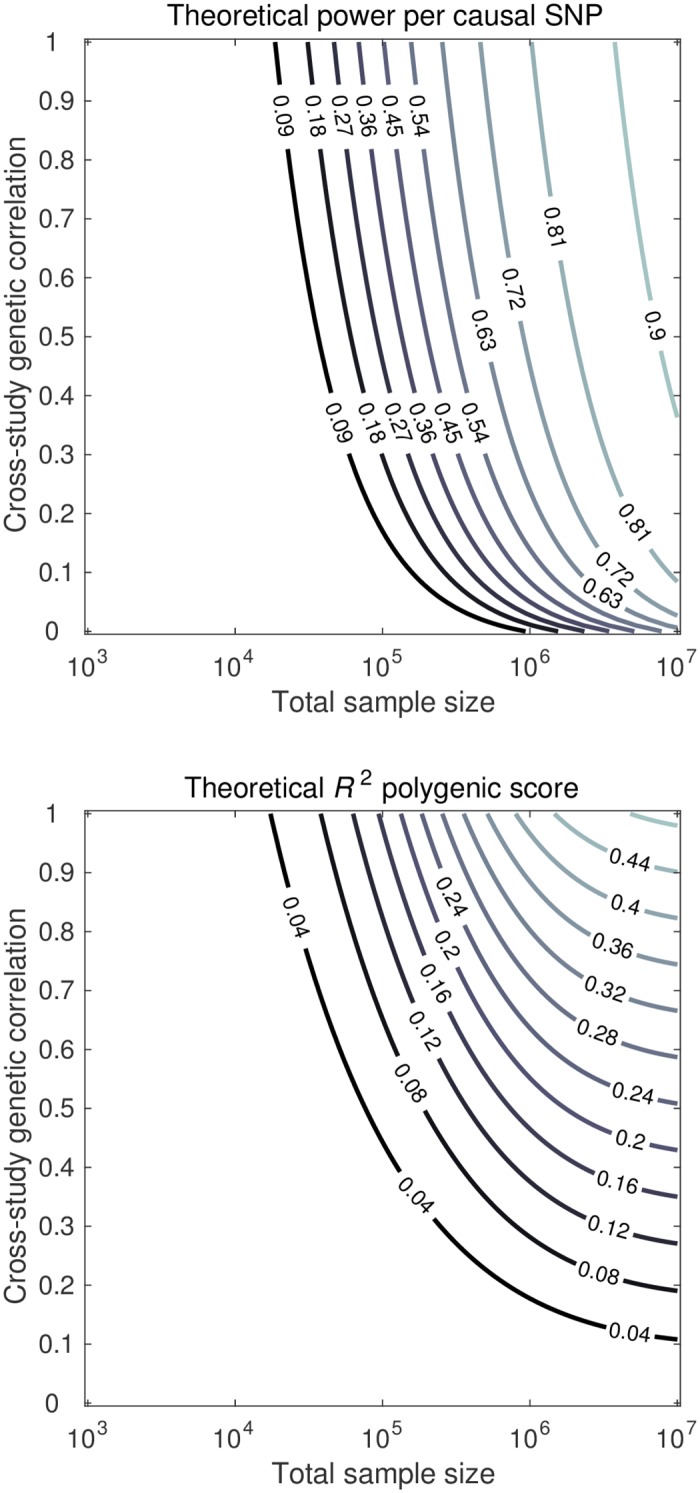
Theoretical predictions of power per causal SNP (upper panel) and out-of-sample *R*^2^ of the PGS (lower panel), for total sample size (*x*-axis) and cross-study genetic correlation (*y*-axis). Factor levels: 50 studies, 100k independent SNPs, and $h_{\text{SNP}}^{2}=50\%$ arising from a subset of 1k independent SNPs.

Fig 1 shows contour plots for the power per truly associated SNP and *R*^2^, for a setting with 50 studies, for a trait with $h_{\text{SNP}}^{2}=50\%$, for various combinations of total sample size and CGR. Increasing total sample size enhances both power and *R*^2^. When the CGR is perfect, power and *R*^2^ (relative to $h_{\text{SNP}}^{2}$) have a near-identical response to sample size. This similarity in response gets distorted when the CGR decreases. For instance, in the scenario of 100k SNPs of which a subset of 1k SNPs is causal with $h_{\text{SNP}}^{2}=50\%$, in a sample of 50 studies with a total sample size of 10 million individuals, a CGR of one yields 94% power per causal SNP and an *R*^2^ of 49%, which is 98% of the SNP heritability, whereas for a CGR of 0.2 the power is still 87% per SNP, while the *R*^2^ of the PGS is 8.5%, which is only 17% of $h_{\text{SNP}}^{2}$. Thus, *R*^2^ is far more sensitive to an imperfect CGR than the meta-analytic power is. This finding is also supported by the approximations of power in Eq 1 and of PGS *R*^2^ in Eq 2; these expressions show that, for two discovery studies, the CGR has a linear effect on the variance of the meta-analysis *Z* statistic, whereas, for one discovery and one hold-out sample, the PGS *R*^2^ is quadratically proportional to the CGR.

#### SNP heritability and CGR

**Fig 2:**
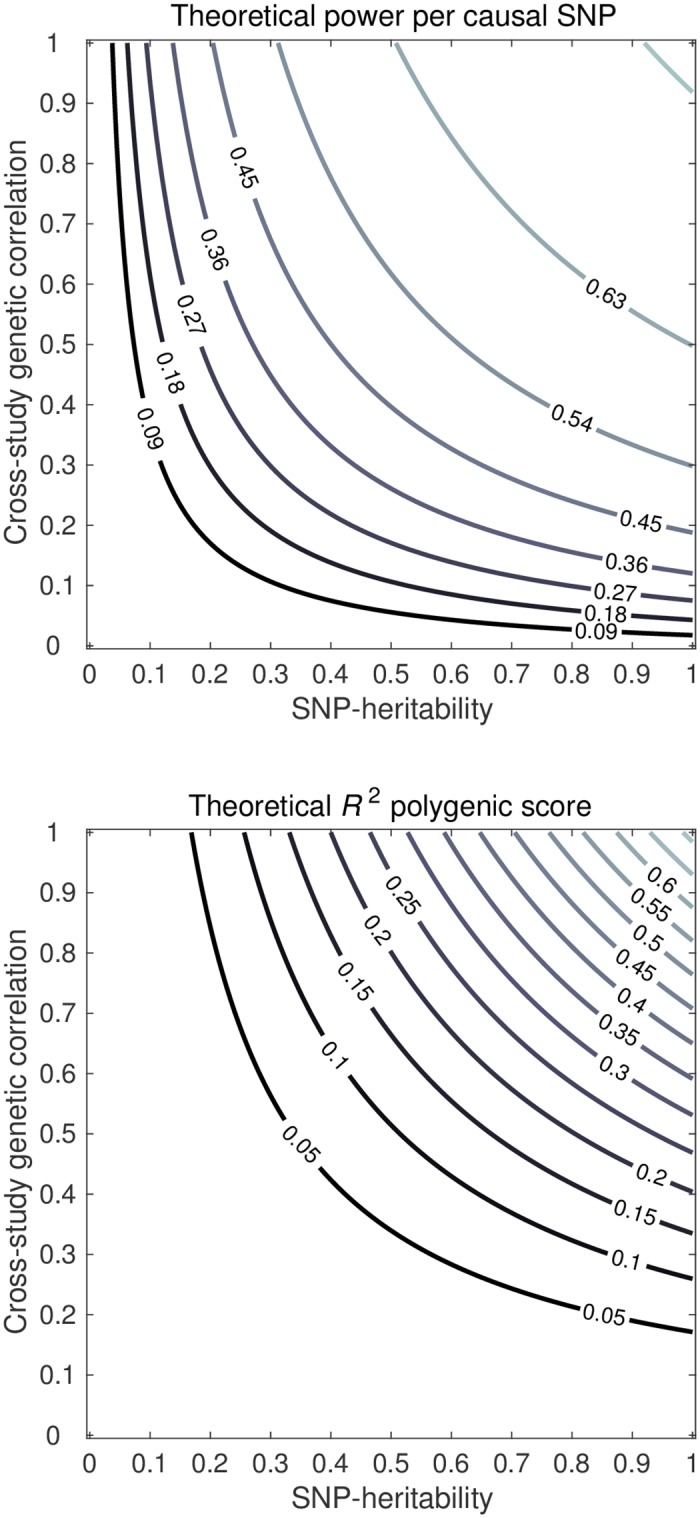
Theoretical predictions of power per causal SNP (upper panel) and out-of-sample *R*^2^ of the PGS (lower panel), for a trait that across studies has SNP heritability (*x*-axis) and cross-study genetic correlation (*y*-axis). Factor levels: 50 studies, sample size 5,000 individuals per study, 100k independent SNPs, and heritability arising from a subset of 1k independent SNPs.

Fig 2 shows contour plots for the power per truly associated SNP and *R*^2^ for a setting with 50 studies, with a total sample size of 250,000 individuals, for 1k causal SNPs and 100k SNPs in total, for various combinations of $h_{\text{SNP}}^{2}$ and CGR. The figure shows a symmetric response of both power and *R*^2^ to CGR and $h_{\text{SNP}}^{2}$. For instance, when $h_{\text{SNP}}^{2}=25\%$ and CGR = 0.5 across all studies, the power is expected to be around 34% and the *R*^2^ 3.0%. When these numbers are interchanged (i.e., $h_{\text{SNP}}^{2}=50\%$ and CGR = 0.25), similarly, the power is expected to be 35% and the *R*^2^ 2.9%. Hence, in terms of both *R*^2^ and power, a low heritability can be compensated by a high CGR (e.g., by means of homogeneous measures across studies) and a low CGR can be compensated by high heritability. When either CGR or heritability is equal to zero, both power and *R*^2^ are decimated in the multi-study setting. However, when both are moderately low but still substantially greater than zero, neither power nor *R*^2^ are completely diminished.

#### Number of studies and CGR

**Fig 3:**
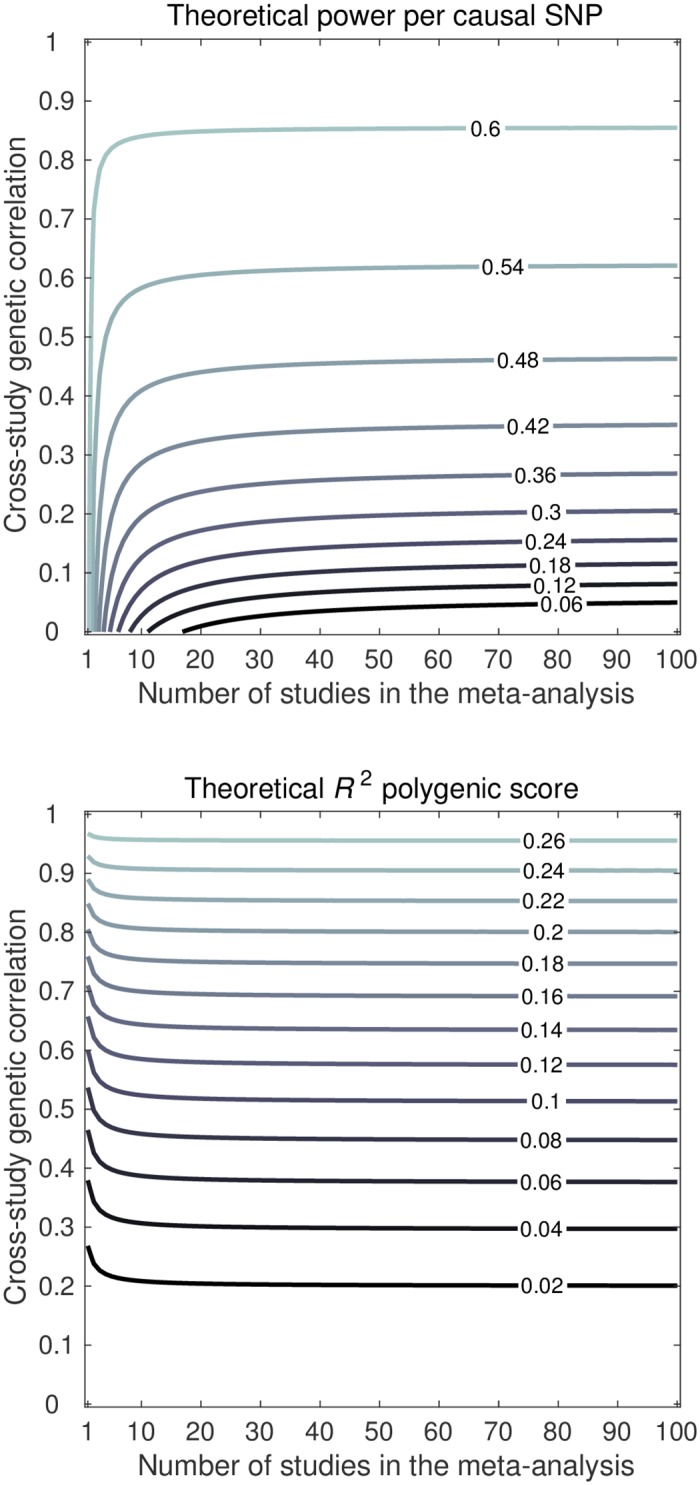
Theoretical predictions of power per causal SNP (upper panel) and out-of-sample *R*^2^ of the PGS (lower panel), for a trait with GWAS results from the number of studies (*x*-axis) with cross-study genetic correlation (*y*-axis). Factor levels: total sample size 250,000 individuals, 100k independent SNPs, and $h_{\text{SNP}}^{2}=50\%$ arising from a subset of 1k independent SNPs.

Fig 3 shows contour plots for the power per truly associated SNP and *R*^2^ for a trait with $h_{\text{SNP}}^{2}=50\%$, 1k causal SNPs, 100k SNPs in total, and a fixed total sample size of 250,000 individuals. In this figure, various combinations of the CGR and the number of studies are considered. Logically, when there is just one study for discovery, CGR does not affect power. However, even for two studies, the effect of CGR on power is quite pronounced. For instance, when CGR is a half, the power per causal SNP is 63% for one study, 58% for two studies, 51% for ten studies, and 50% for 100 studies. Thus, when the number of studies is low, increasing the number of studies makes the effect of CGR on power more pronounced rapidly. When the number of studies is large, further increases in the number of studies hardly make the effect of CGR on power more pronounced.

For a given number of studies, we observed that the effect CGR has on *R*^2^ is stronger than the effect it has on power. This observation is in line with the approximated theoretical *R*^2^ in Eq 2, indicating that *R*^2^ is quadratically proportional to CGR. However, an interesting observation is that this quadratic relation lessens as the number of studies grows large, despite the total sample size being fixed. For instance, at a CGR of a half, the *R*^2^ in the hold-out sample is expected to be 6.9% when there is only one discovery study. However, the expected *R*^2^ is 8.1% for two discovery studies, 9.3% for ten discovery studies, and 9.6% for 100 discovery studies. A likely reason for this pattern is that, in case of one discovery study, the PGS is influenced relatively strongly by the study-specific component of the genetic effects. This idiosyncrasy is not of relevance for the hold-out sample. As the number of studies increases—even though each study brings its own idiosyncratic contribution—each study consistently conveys information about the part of the genetic architecture which is common across the studies. Since the idiosyncratic contributions from the studies are independent, they tend to average each other out, whereas the common underlying architecture gets more pronounced as the number of studies in the discovery increases, even if the total sample size is fixed.

#### SNP heritability in the hold-out sample

**Fig 4:**
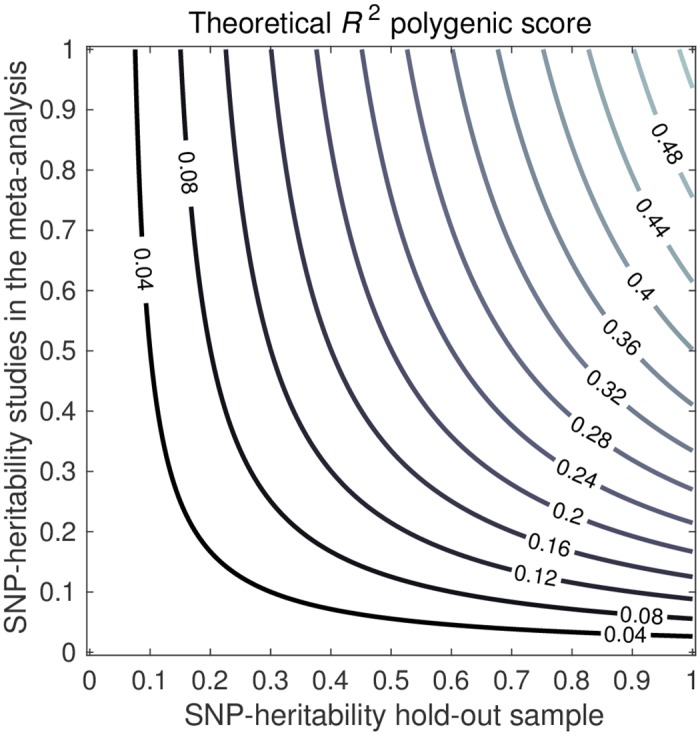
Theoretical predictions of out-of-sample *R*^2^ of the PGS, for the SNP heritability in the hold-out sample (*x*-axis) and the SNP heritability in the discovery samples (*y*-axis). Factor levels: 50 studies, sample size 5,000 individuals per study, cross-study genetic correlation 0.8, 100k independent SNPs, and heritability arising from a subset of 1k independent SNPs.

Fig 4 shows a contour plot for the PGS *R*^2^ based on a meta-analysis of 50 studies with a total sample size of 250,000 individuals, with 1k causal SNPs and 100k SNPs in total, and a CGR of 0.8 between both the discovery studies and the hold-out sample. In the plot, various combinations of $h_{\text{SNP}}^{2}$ in the discovery samples and $h_{\text{SNP}}^{2}$ in the hold-out sample are considered. The response of PGS *R*^2^ to heritability in the discovery sample and the hold-out sample is quite symmetric, in the sense that a low $h_{\text{SNP}}^{2}$ in the discovery samples and a high $h_{\text{SNP}}^{2}$ in the hold-out sample yield a similar *R*^2^ as a high $h_{\text{SNP}}^{2}$ in the discovery sample and a low $h_{\text{SNP}}^{2}$ in the hold-out sample. However, *R*^2^ is slightly more sensitive to $h_{\text{SNP}}^{2}$ in the hold-out sample than in the discovery samples. For instance, when SNP heritability in the discovery samples is 50% and 25% in the hold-out sample, the expected *R*^2^ is 10%, whereas in case the SNP heritability is 25% in the discovery samples and 50% in the hold-out sample, the expected *R*^2^ is 13%.

#### CGR between sets of studies

**Fig 5:**
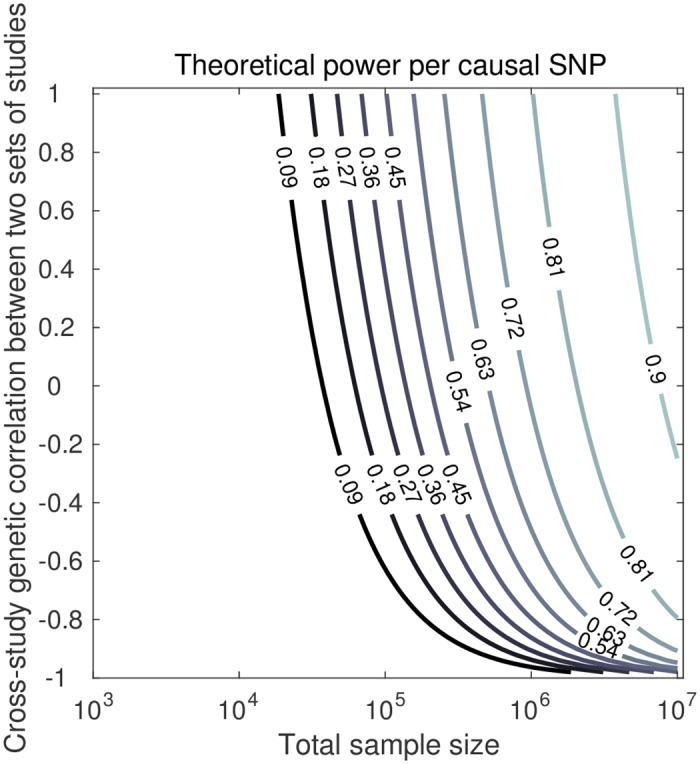
Theoretical predictions of power per causal SNP, for total sample size (*x*-axis) and CGR between two sets of studies (*y*-axis). Factor levels: 2 sets of 50 studies, CGR equal to 1 within both sets, 100k independent SNPs, and $h_{\text{SNP}}^{2}=50\%$ arising from a subset of 1k independent SNPs.

Fig 5 shows a contour plot for the power per truly associated SNP in a setting where there are two sets consisting of 50 studies each. Within each set, the CGR is equal to one, whereas between sets the CGR is imperfect. Consider, for example, a scenario where one wants to meta-analyze GWAS results for height from a combination of two sets of studies; one set of studies consisting primarily of individuals of European ancestry and one set of studies with mostly individuals of Asian ancestry in it. Now, one would expect CGRs close to one between studies consisting primarily of individuals of European ancestry and the same for the CGRs between studies consisting primarily of individuals of Asian ancestry. However, the CGRs between those two sets of studies may be less than one.

As is shown in S1 Derivations, in case the CGR between the two sets of studies, $C_{1}$ and $C_{2}$, is zero, meta-analyzing the two sets jointly yields power $\beta_{C_{1}\cup \;C_{2}}\leq \max\{\beta_{C_{1}},\beta_{C_{2}}\}$ and $\beta_{C_{1}\cup \;C_{2}}\geq \min\{\beta_{C_{1}},\beta_{C_{2}}\}$, where $\beta_{A}$ denotes the power in set of studies A. In particular, when $\beta_{C_{1}}=\beta_{C_{2}}$ we have under a CGR of zero between the sets, that $\beta_{C_{1}\cup \;C_{2}}=\beta_{C_{1}}=\beta_{C_{2}}$. Since in Fig 5 we considered two equally-powered sets, the power of a meta-analysis using both sets, under zero CGR between sets, is identical to the power obtained when meta-analyzing, for instance, only the first set. However, as CGR between sets increases, so does power. For instance, when a total sample size of 250,000 individuals is spread across 2 clusters, each cluster consisting of 50 studies (i.e., sample size of 125,000 individuals per cluster and 2,500 individuals per study), under $h_{\text{SNP}}^{2}=50\%$ due to 1k causal SNPs, a CGR of one within each cluster, and CGR of zero between clusters, the power is expected to be 49%, which is identical to the power of a meta-analysis of either the first or the second cluster. However, if the CGR between clusters is 0.5 instead of zero, the power goes up to 58%. In terms of the expected number of hits, this cross-ancestry meta-analysis yields an expected 82 additional hits, compared to a meta-analysis considering only one ancestry.

Alternatively, one could carry out a meta-analysis in each set of studies and pool the hits across these sets. However, this would imply more independent tests being carried out, and, hence, the need for a more stringent genome-wide significance threshold, in order to keep the false-positive rate fixed. Therefore, this route may yield less statistical power than a meta-analysis of merely one of the two sets or a joint analysis of both. Ideally, in the scenario where between-population heterogeneity is likely, one should apply a meta-analysis method that accounts for the heterogeneity (e.g., [26–31]). By applying such a method, one can consider all GWAS results from different ancestry groups in one analysis.

### Empirical results for SNP-based heritability and CGR

In Table 1 we report univariate GREML estimates of SNP heritability and bivariate GREML estimates of genetic correlation for traits that attained a pooled sample size of at least 18,000 individuals, which gave us at least 50% power to detect a genetic correlation near one for a trait that has a SNP heritability of 10% or more [45]. The smallest total sample size is *N_T_* = 19,184 for self-rated health. Details per phenotype on sample size, univariate estimates of SNP heritability, and bivariate estimates of genetic correlation, stratified across studies, and cross-study averages, are provided in S1 Table. Results stratified across sexes are listed in S2 Table.

**Table 1:** GREML estimates of SNP heritability and genetic correlation across studies and sexes.

Phenotype	*N*	Estimates SNP heritability^1^			Estimates genetic correlation^1^^,^^2^			
		pooled^3^	study^4^	sexes^5^	RS–STR	RS–HRS	STR–HRS	Females–Males
Height	20,458	43.3% (1.8%) ***	44.9%	44.0%	0.976 (0.102) ***	0.954 (0.095) ***	0.967 (0.106) ***	0.981 (0.067) ***
BMI	20,449	20.9% (1.7%) ***	21.9%	22.8%	1.000 (0.269) ***	0.914 (0.172) ***	0.847 (0.246) ***	0.794 (0.122) *** †
*EduYears*	20,619	16.4% (1.7%) ***	18.2%	18.4%	0.690 (0.233) ***	0.659 (0.224) *** †	1.000 (0.263) ***	0.832 (0.162) ***
*CurrCigt*	20,686	18.2% (4.0%) ***	19.1%	24.2%	1.000 (0.643) ***	0.611 (0.448) *	1.000 (0.607) ***	0.543 (0.257) *** †
*CurrDrinkFreq*	20,072	7.0% (2.6%) ***	10.3%	8.3%	1.000 (0.666) ***	0.298 (0.670)	-0.056 (0.647)	1.000 (2.068) *
Self-rated health	19,184	10.3% (1.8%) ***	15.7%	9.5%	0.626 (0.439) **	0.363 (0.223) ** ††	0.447 (0.278) **	1.000 (0.349) ***

^1^ Standard errors between parentheses.

^2^ Significance of deviations from one only tested for genetic correlations.

^3^ Univariate estimates from pooled data.

^4^ Sample-size weighted averages of univariate estimates across studies.

^5^ Sample-size weighted averages of univariate estimates across sexes.

* > 0 at 10% sign.

** > 0 at 5% sign.

*** > 0 at 1% sign.

^†^ < 1 at 10% sign.

^††^ < 1 at 5% sign.

^†††^ < 1 at 1% sign.

The univariate estimates of SNP heritability based on the pooled data assume perfect CGRs. Therefore, such estimates of SNP heritability are downwards biased when based on data from multiple studies with imperfect CGRs. To circumvent this bias, we estimated SNP heritability in each study separately, and focused on the sample-size-weighted cross-study average estimate of SNP heritability.

For both height and BMI, we observed genetic correlations close to one across pairs of studies and between females and males. For years of schooling (*EduYears*) we found a CGR around 0.8 when averaged across pairs of studies. Similarly, the genetic correlation for *EduYears* in females and males lies around 0.8. The CGR of self-rated health is substantially below one across the pairs of studies, whilst the genetic correlation between females and males seems to lie around one. The reason for this difference in the genetic correlation of self-rated health between pairs of studies and between females and males may be due to the difference in the questionnaire across studies, discussed in S1 Data. The questionnaire differences can yield a low CGR, while not precluding the remaining genetic overlap for this measure across the three studies, to be highly similar for females and males. For *CurrCigt* and *CurrDrinkFreq*, the estimates of CGR and of genetic correlation between females and males are non-informative. For these two traits the standard errors of the genetic correlations estimates are large, mostly greater than 0.5. In addition, for *CurrDrinkFreq* there is strong volatility in the CGR estimate across pairs of studies.

### Attenuation in power and *R*^2^ due to imperfect CGR

Considering only the traits for which we obtained accurate estimates of CGR and SNP heritability (i.e., with low standard errors), we used the MetaGAP calculator to predict the number of hits in a set of discovery samples and the PGS *R*^2^ in a hold-out sample, in prominent GWAS efforts for these traits. Details and notes on the results from existing studies, used as input for the MetaGAP calculations, can be found S3 Table. Importantly, as reported in S4 Table, for the traits under consideration here, large-scale GWAS results to date have been obtained using fixed-effects meta-analyses.

Since we only had accurate estimates for height, BMI, *EduYears*, and self-rated health, we focused on these four phenotypes. For these traits, we computed sample-size-weighted average CGR estimates across the pairs of studies. Table 2 shows the number of hits and PGS *R*^2^ reported in the most comprehensive GWAS efforts to date for the traits of interest, together with predictions from the MetaGAP calculator. We tried several values for the number of independent haplotype blocks (i.e., 100k, 150k, 200k, 250k) and for the number of trait-associated blocks (i.e., 10k, 15k, 20k, 25k). Overall, 250k blocks of which 20k trait-affecting yielded theoretical predictions in best agreement with the empirical observations; we acknowledge the potential for some overfitting (i.e., two free parameters set on the basis of 17 data points; 10 data points for the reported number of hits and 7 for PGS *R*^2^).

**Table 2:** Predicted and observed number of genome-wide-significant hits and PGS *R*^2^, for large-scale GWAS efforts to date for height, BMI, *EduYears*, and self-rated health, assuming 250k effective SNPs (i.e., independent haplotype blocks) of which 20k trait-affecting, using averaged GREML estimates from Table 1 for setting SNP heritability and CGR. Notes on the sources for the large-scale GWAS efforts are listed in S3 Table.

Phenotype	Main studies			Architecture		Number of hits				PGS *R*^2^ using all SNPs			
	Study	*N*	*C* **	$h_{\text{SNP}}^{2}$	CGR	Study	Theory|CGR		Attenuation*	Study	Theory|CGR		Attenuation*
							<1	=1			<1	=1	
Height	Wood *et al*. (2014) [1]	253,288	79	44.9%	0.965	697	647.26	700.24	8%	13.5%	13.2%	14.0%	6%
	Allen *et al*. (2010) [46]	183,727	61	44.9%	0.965	180	292.03	320.77	9%	10.0%	10.5%	11.1%	6%
	Weedon *et al*. (2008) [47]	13,665	5	44.9%	0.965	7	0.00	0.00	*n.a.*	2.9%	1.0%	1.1%	7%
BMI	Locke *et al*. (2015) [2]	339,224	125	21.9%	0.917	97	188.52	241.07	22%	6.5%	4.3%	5.0%	14%
	Speliotes *et al*. (2010) [48]	123,865	46	21.9%	0.917	19	5.48	7.64	28%	2.5%	1.8%	2.1%	15%
	Willer *et al*. (2008) [49]	32,387	15	21.9%	0.917	1	0.01	0.02	65%	*n.a.*	0.5%	0.6%	16%
*EduYears*	Okbay *et al*. (2016) [7]	405,072	65	18.2%	0.783	162	115.28	235.90	51%	*n.a.*	2.7%	4.1%	36%
	Okbay *et al*. (2016) [7]	293,723	64	18.2%	0.783	74	39.30	88.93	56%	3.9%	2.0%	3.2%	36%
	Rietveld *et al*. (2013) [50]	101,069	42	18.2%	0.783	1	0.63	1.64	62%	2.5%	0.8%	1.2%	38%
Self-rated health	Harris *et al*. (2016) [51]	111,749	1	15.7%	0.468	13	1.35	1.35	0%	*n.a.*	0.2%	1.0%	78%

* Attenuation measures the relatively loss in expected power and *R*^2^ due to a CGR in accordance with averaged GREML estimates from Table 1.

** *C* denotes the number of studies in the meta-analysis.

For height—the trait with the lowest standard error in the estimates of $h_{\text{SNP}}^{2}$ and CGR—the predictions of the number of hits and PGS *R*^2^ for the two largest GWAS efforts are much in line with theoretical predictions. For the smaller GWAS of 13,665 individuals [47], our estimates seem slightly conservative; 0 hits expected versus the 7 reported. However, in our framework, we assumed that each causal SNP has the same *R*^2^. Provided there are some differences in *R*^2^ between causal SNPs, the first SNPs that are likely to reach genome-wide significance in relatively small samples, are the ones with a comparatively large *R*^2^. This view is supported by the fact that a PGS based on merely 20 SNPs already explains 2.9% of the variation in height. Hence, for relatively small samples our theoretical predictions of power and *R*^2^ may be somewhat conservative. In addition, the 10k SNPs with the lowest meta-analysis *p*-values can explain about 60% of the SNP heritability [1]. If the SNPs tagging the remaining 40% each have similar predictive power as the SNPs tagging the first 60%, then the number of SNPs needed to capture the full $h_{\text{SNP}}^{2}$ would lie around 10k/0.6 = 17k, which is somewhat lower than the 20k which yields the most accurate theoretical predictions. However, as indicated before, the SNPs which appear most prominent in a GWAS are likely to be the ones with a greater than average predictive power. Therefore, the remaining 40% of $h_{\text{SNP}}^{2}$ is likely to be stemming for SNPs with somewhat lower predictive power. Hence, 20k associated independent SNPs is not an unreasonable number for height.

The notion of a GWAS first picking up the SNPs with a relatively high *R*^2^ is also supported by the predicted and observed number of hits for the reported self-rated-health GWAS [51]; given a SNP heritability estimate between 10% [51] and 16% (Table 2), according to our theoretical predictions, a GWAS in a sample of around 110k individuals is unlikely to yield even a single genome-wide significant hit. Nevertheless, this GWAS has yielded 13 independent hits. This finding supports the idea that for various traits, some SNPs with a relatively high *R*^2^ are present. However, there is uncertainty in the number of truly associated loci. More accurate estimates of this number may improve the accuracy of our theoretical predictions.

For BMI our predictions of PGS *R*^2^ were quite in line with empirical results. However, for the number of hits, our predictions for the largest efforts seemed overly optimistic. We therefore suspect that the number of independent SNPs associated with BMI is higher than 20k; a higher number of associated SNPs would reduce the GWAS power, while preserving PGS *R*^2^, yielding good agreement with empirical observation. Nevertheless, given the limited number of data points, this strategy of setting the number of causal SNPs would increase the chance of overfitting.

For *EduYears* we observed that the reported number of hits is in between the expected number of hits when the CGR is set to the averaged GREML estimate of 0.783 and when the CGR is set to one. Given the standard errors in the CGR estimates for *EduYears*, the CGR might very well be somewhat greater than 0.783, which would yield a good fit with the reported number of hits. However, as with the number of truly associated SNPs for BMI, in light of the risk of overfitting, we can make no strong claims about a slightly higher CGR of *EduYears*.

Overall, our theoretical predictions of the number of hits and PGS *R*^2^ are in moderate agreement with empirical observations, especially when bearing in mind that we are looking at a limited number of data points, making chance perturbations from expectation likely. In addition, regarding the number of hits, the listed studies are not identical in terms of the procedure to obtain the independent hits. Therefore, the numbers could have been slightly different, had the same pruning procedure been used across all reported studies.

Regarding attenuation, we observed a substantial spread in the predicted number of hits and PGS *R*^2^ when assuming either a CGR equal to one, or a CGR in accordance with empirical estimates, with traits with lower CGR suffering from stronger attenuation in power and predictive accuracy. In line with theory, *R*^2^ falls approximately quadratically with CGR. For instance, for self-rated health, the estimated CGR of about 0.5, would yield a PGS that retains approximately 0.5^2^ = 25% of the *R*^2^ it would have had under a CGR of one. Hence the approximated attenuation is 75%. This approximation is corroborated by the theoretical relative attenuation of 78%.

Given our CGR estimates, the theoretical relative loss in PGS *R*^2^ is 6% for height, 14% for BMI, 36% for *EduYears*, and 78% for self-rated health, when compared to the *R*^2^ of PGSs under perfect CGRs (Table 2). These losses in *R*^2^ are unlikely to be reduced by larger sample sizes and denser genotyping.

Somewhat contrary to expectation, the number of hits seems to respond even more strongly to CGR than PGS *R*^2^. However, since in each study under consideration the average power per associated SNP is quite small, a small decrease in power per SNP in absolute terms can constitute a substantial decrease in relative terms. For instance, when one has 2% power per truly associated SNP, an absolute decrease of 1%—leaving 1% power—constitutes a relative decrease of 50% of power per causal SNP, and thereby a 50% decrease in the expected number of hits. This strong response shows, for example, in the case of *EduYears*, where the expected number of hits drop by about 37% when going from a CGR of one down to a CGR of 0.783.

## Discussion

We have shown that imperfect cross-study genetic correlations (CGRs) are likely to contribute to the gap between the phenotypic variation accounted for by all SNPs jointly and by the leading GWAS efforts to date. We arrived at this conclusion in five steps. First, we developed a Meta-GWAS Accuracy and Power (MetaGAP) calculator that accounts for the CGR. This online calculator relates the statistical power to detect associated SNPs and the *R*^2^ of the polygenic score (PGS) in a hold-out sample to the number of studies, sample size and SNP heritability per study, and the CGR. The underlying theory shows that there is a quadratic response of the PGS *R*^2^ to CGR. Moreover, we showed that the power per associated SNP is also affected by CGR.

Second, we used simulations to demonstrate that our theory is robust to several violations of the assumptions about the underlying data-generating process, regarding the relation between allele frequency and effect size, the distribution of allele frequencies, and the factors contributing to CGR. Further research needs to assess whether our theoretical predictions are also accurate under an even broader set of scenarios (e.g., when studying a binary trait).

Third, we used a sample of unrelated individuals from the Rotterdam Study, the Swedish Twin Registry, and the Health and Retirement Study, to estimate SNP-based heritability as well as the CGR for traits such as height and BMI. Although our CGR estimates have considerable standard errors, the estimates make it likely that for many polygenic traits the CGR is positive, albeit smaller than one.

Fourth, based on these empirical estimates of SNP heritability and CGR for height, BMI, years of education, and self-rated health, we used the MetaGAP calculator to predict the number of expected hits and the expected PGS *R*^2^ for the most prominent studies to date for these traits. We found that our predictions are in moderate agreement with empirical observations. Our theory seems slightly conservative for smaller GWAS samples. For large-scale GWAS efforts our predictions were in line with the outcomes of these efforts. More accurate estimates of the number of truly associated loci may further improve the accuracy of our theoretical predictions.

Fifth, we used our theoretical model to assess statistical power and predictive accuracy for these GWAS efforts, had the CGR been equal to one for the traits under consideration. Our estimates of power and predictive accuracy in this scenario indicated a strong decrease in the PGS *R*^2^ and the expected number of hits, due to imperfect CGRs. Though these observations are in line with expectation for predictive accuracy, for statistical power the effect was larger than we anticipated. This finding can be explained, however, by the fact that though the absolute decrease in power per SNP is small, the relative decrease is large, since the statistical power per associated SNP is often low to begin with.

Overall, our study affirms that although PGS accuracy improves substantially with further increasing sample sizes, in the end PGS *R*^2^ will continue to fall short of the full SNP-based heritability. Hence, this study contributes to the understanding of the hiding heritability reported in the GWAS literature.

Regarding the etiology of imperfect CGRs, the likely reasons are heterogeneous phenotype measures across studies, gene–environment interactions with underlying environmental factors differing across studies, and gene–gene interactions where the average effects differ across studies due to differences in allele frequencies. Our study is not able to disentangle these different causes; by estimating the CGR for different traits we merely quantify the joint effect these three candidates have on the respective traits.

However, in certain situations it may be possible to disentangle the etiology of imperfect CGRs to some extent. For instance, in case one considers a specific phenotype that is usually studied by means of a commonly available but relatively heterogeneous and/or noisy measure, while there also exists a less readily available but more accurate and homogeneous measure. If one has access to both these measures in several studies, one can compare the CGR estimates for the more accurate measure and the CGR estimates for the less accurate but more commonly available measure. Such a comparison would help to disentangle the contribution of phenotypic heterogeneity and genetic heterogeneity to the CGR of the more commonly available measure.

In considering how to properly address imperfect CGRs, it is important to note that having a small set of large studies, rather than a large set of small studies, does not necessarily abate the problem of imperfect genetic correlations. Despite the fact that having fewer studies can help to reduce the effects of heterogeneous phenotype measures, larger studies are more likely to sample individuals from different environments. If gene–environment interactions do play a role, strong differences in environment between subsets of individuals in a study can lead to imperfect genetic correlations within that study. The attenuation in power and accuracy resulting from such within-study heterogeneity may be harder to address than cross-study heterogeneity.

Our findings stress the importance of considering the use of more sophisticated meta-analysis methods that account for cross-study heterogeneity [26–31]. We believe that the online MetaGAP calculator will prove to be an important tool for assessing whether an intended fixed-effects meta-analysis of GWAS results from different studies is likely to yield meaningful outcomes.

## Supporting Information

S1 Derivations Statistical power of a meta-analysis of GWAS results. (PDF)

S2 Derivations Predictive accuracy of a polygenic score based on SNP-effect estimates from a meta-analysis of GWAS results. (PDF)

S1 Note Genetic correlations. (PDF)

S1 Simulations Assessment of the accuracy of the MetaGAP calculator. (PDF)

S1 Data Description of genotype and phenotype data, and quality control. (PDF)

S1 Estimation GREML estimation procedure. (PDF)

S1 Table GREML estimates of SNP heritability and genetic correlation across studies. (PDF)

S2 Table GREML estimates of SNP heritability and genetic correlation across sexes. (PDF)

S3 Table Details and notes on the sources for the sample size, the number of meta-analyzed studies, the number of genome-wide-significant hits, and the PGS *R*^2^, for large-scale GWAS efforts to date for height, BMI, *EduYears*, and self-rated health, reported in the same order as in Table 2. (PDF)

S4 Table Meta-analysis methods used in large-scale GWAS efforts to date for height, BMI, *EduYears*, and self-rated health, reported in the same order as in Table 2. (PDF)
