# Supplementary material for: Meta-GWAS Accuracy and Power (MetaGAP) Calculator Shows that Hiding Heritability Is Partially Due to Imperfect Genetic Correlations across Studies
Source: PLoS Genet. 2017 Jan 17;13(1):e1006495. doi: 10.1371/journal.pgen.1006495 (PMC5240919; doi:10.1371/journal.pgen.1006495)
Supplement: S1 Derivations — (PDF) [file pgen.1006495.s001.pdf]

S1 Derivations. Statistical power of a meta-analysis of GWAS results.

In this supporting information section, we derive an expression for the power of a meta-analysis of GWAS results, under a design with many studies, with arbitrary sample sizes, SNP-based heritability, and cross-study genetic correlation (CGR).

First, the underlying assumptions are presented. Second, we write the GWAS Z statistics in terms of the true SNP effect and noise. Third, we incorporate cross-study genetic correlations by assuming a model with random SNP effects that are correlated imperfectly across studies. Using the Cholesky decomposition of the cross-study genetic correlation matrix, we write the correlated SNP effects in terms of a weighted sum of independent genetic factors. By means of this decomposition into independent factors, we derive the distribution of the Z statistic in a given study, as well as the distribution of the multi-study meta-analysis Z statistic. From the latter distribution we obtain a framework for performing multi-study power calculations.

It is important to note that models which incorporate random SNP effects have been widely used, for instance, to estimate variance components [1] and genetic correlations across traits and samples [2], to control for cryptic relatedness and population structure in a GWAS [3], and to distill the constituents of genomic inflation [4, 5]. Hence, the novelty in our work lies not in using random SNP-effect models to incorporate imperfect genetic correlations across studies. Instead the novelty lies in the subsequent step, *viz.*, to use such models in order to perform power calculations under the presence of imperfect CGRs.

Assumptions We derive an expression of statistical power for a quantitative trait in sample-size weighted meta-analysis [6]. In order to arrive at a tractable expression of statistical power, we make the following assumptions.

1. When considering a given SNP in the GWAS, any phenotypic variance due to other SNPs gets absorbed by the normally, independent, and identically distributed residual term (which is what happens when studying a sample of unrelated individuals, and which is in line with assumptions underlying most GWAS packages, except for family-based and mixed-linear-model-type GWAS software). This assumption keeps the algebra simple at the cost of a small loss in generality. In S1 Simulations we show that violations of this assumption do not affect results.

2. The regressors (i.e., SNP data) in the meta-analysis studies are fixed (i.e., non-stochastic)—this assumption is equivalent to conditioning on the genotype data. This assumption also keeps the algebra simple at the cost of a small loss in generality. In S1 Simulations we show that violations of this assumption do not affect results.

3. Each causal locus is shared across all studies. This assumption enables us to consider CGRs as a one-dimensional factor that is shaped solely by the cross-study correlation of the effects of trait-affecting haplotype blocks. In S1 Simulations we show that violations of this assumption hardly affect results.

4. The genome can be divided into independent haplotype blocks, where for each block we have precisely one SNP that tags all the variation within this block. By means of this assumption, we can ignore linkage disequilibrium, thereby strongly reducing the complexity of our derivations. In addition, we assume that the effects of trait-affecting haplotype blocks are independent. The former assumption would imply that all trait-affecting variation in a haplotype block can be captured by the single tag SNP for that block. Although we make no claim that common SNPs perfectly tag all trait affecting variants, we do claim that a relatively small set of common SNPs can tag the heritability as estimated using common SNPs. Consequently, when using estimates of SNP heritability based on common SNPs, we deem this assumption and its implications to generate little bias in our theoretical predictions.

5. The effect sizes of SNPs are inversely related to SNP variance (i.e., rare variants have larger effects than common variants, such that the expected R^2 of each causal SNP, with respect to the phenotype, is equal regardless of allele frequency). This assumption makes it possible to compute statistical power without having to specify the allele frequency and an *a priori* unknown effect size. Under this assumption, SNP heritability and the number of trait-affecting haplotype blocks replace a SNP-specific effect size and allele frequency. In S1 Simulations we show that violations of this assumption hardly affect results.

Single-SNP model Here, we write the GWAS Z statistic in a given study for a given SNP, as a function of the true effect and noise. This decomposition into true effect and noise helps to derive the distribution of the Z statistic.

For studies $j = 1, \ldots, C$ and SNPs $k = 1, \ldots, S$, let the model for a quantitative trait with a single SNP as predictor (Assumption 1) for the mean-centered phenotype $\mathbf{y}_j$ be given by

$$\mathbf{y}_j = \mathbf{x}_{jk}\beta_{jk} + \boldsymbol{\varepsilon}_j, \tag{1}$$

$$\boldsymbol{\varepsilon}_j \sim \mathcal{N}\left(\mathbf{0}, \sigma^2_{\varepsilon_j}\mathbf{I}_{N_j}\right) \tag{2}$$

where $\mathbf{x}_{jk}$ denotes the mean-centered genotype vector of SNP k in study j, scaled such that $(\mathbf{x}_{jk}^\top\mathbf{x}_{jk})/N_j = 1$. In Eq. 1, β_{jk} is the effect of SNP k in study j. In Eq. 2, $\boldsymbol{\varepsilon}_j$ is the residual and $\mathbf{I}_{N_j}$ the $N_j \times N_j$ identity matrix, where N_j is the sample size of study j.

The GWAS estimate of β_{jk} for a quantitative trait is usually obtained by applying OLS. Hence, it can be written

as

$$\widehat{\beta}_{jk} = \left(\frac{1}{N_j}\mathbf{x}_{jk}^{\top}\mathbf{x}_{jk}\right)^{-1}\frac{1}{N_j}\mathbf{x}_{jk}^{\top}\mathbf{y}_j \tag{3}$$

$$= \frac{1}{N_j}\mathbf{x}_{jk}^{\top}\mathbf{y}_j \tag{4}$$

$$= \frac{1}{N_j}\mathbf{x}_{jk}^{\top}\mathbf{x}_{jk}\beta_{jk} + \frac{1}{N_j}\mathbf{x}_{jk}^{\top}\boldsymbol{\varepsilon}_j \tag{5}$$

$$= \beta_{jk} + \frac{1}{N_j}\mathbf{x}_{jk}^{\top}\boldsymbol{\varepsilon}_j. \tag{6}$$

Using standard results from regression theory assuming fixed regressors (Assumption 2) and the aforementioned scaling of the genotype vector, the theoretical variance of the OLS-estimate of the SNP effect is given by

$$\mathrm{Var}\left(\widehat{\beta}_{jk}\right) = \sigma^2_{\boldsymbol{\varepsilon}_j}\left(\mathbf{x}_{jk}^{\top}\mathbf{x}_{jk}\right)^{-1}$$
$$= \frac{\sigma^2_{\boldsymbol{\varepsilon}_j}}{N_j}.$$

Therefore, the standard error of the OLS estimate is given by

$$\mathrm{s.d.}\left(\widehat{\beta}_{jk}\right) = \frac{\sigma_{\boldsymbol{\varepsilon}_j}}{\sqrt{N_j}}. \tag{7}$$

By taking the ratio of Eq. 6 and 7 we obtain the Z statistic (instead of the commonly used and highly similar t-test statistics) for SNP k in study j. That is,

$$Z_{jk} = \frac{\widehat{\beta}_{jk}}{\mathrm{s.d.}\left(\widehat{\beta}_{jk}\right)} \tag{8}$$

$$= \frac{\sqrt{N_j}}{\sigma_{\boldsymbol{\varepsilon}_j}}\beta_{jk} + \frac{\mathbf{x}_{jk}^{\top}\boldsymbol{\varepsilon}_j}{\sigma_{\boldsymbol{\varepsilon}_j}\sqrt{N_j}}. \tag{9}$$

Let v_{jk} denote the last term in the right-hand side of Eq. 9. Under the aforementioned scaling of the regressor and the distribution of $\boldsymbol{\varepsilon}_j$, it follows from standard properties of the multivariate normal distribution that $v_{jk} \sim \mathcal{N}(0,1)$.

Modelling cross-study genetic correlation We incorporate cross-study genetic correlations by considering a model with random SNP effects, correlated across studies. For ease of derivations, we assume that each causal SNP contributes across all studies (Assumption 3). In order to simplify further derivations, we use a Cholesky decomposition to write correlated SNP effects in terms of independent underlying factors. Using this independent-factor representation, we derive the distribution of a GWAS Z statistic, in terms of the study-specific noise and contributions of the underlying genetic factors.

Genetic correlation can be conceptualized as the correlation between SNP effects across different strata (e.g., across populations, studies, age groups, etc.). Taking studies as 'strata', a group of C studies has $C \times C$ genetic correlation matrix, denoted by $\boldsymbol{P}_{\mathbf{G}}$.

When effects are normally distributed, a given correlation structure between effects is most straightforwardly obtained by constructing the Cholesky decomposition of the correlation matrix, and multiplying independent standard-normal random variables by this decomposition. An interpretation of this decomposition is that it provides a set of weights that transform a set of independent underlying genetic factors into correlated genetic effects.

First, we formalize how to transform independent standard-normal random variables into correlated normal random variables. Let $\mathbf{\Gamma}_{\mathbf{G}}$ be the lower-triangular Cholesky decomposition of the genetic correlation matrix, such that $\mathbf{\Gamma}_{\mathbf{G}}\mathbf{\Gamma}_{\mathbf{G}}^{\top} = \boldsymbol{P}_{\mathbf{G}}$, let $\mathcal{M}$ denote the set of M causal SNPs, let $\mathbf{E}$ be an $C \times M$ matrix of independent standard normal draws from different genetic factors (rows) for the different causal SNPs (columns), and let $\boldsymbol{\eta}_k$ be the column of $\mathbf{E}$ corresponding to causal SNP k. Then

$$\boldsymbol{\eta}_k = \begin{pmatrix} \eta_{1k} \\ \vdots \\ \eta_{Ck} \end{pmatrix} \sim \mathcal{N}\left(\mathbf{0}, \mathbf{I}_C\right),$$

where $\boldsymbol{\eta}_k$ is independent of $\boldsymbol{\eta}_l$ for $l \neq k$ (Assumption 4). Now, for SNP k in the set of causal SNPs, we can define the vector of effects across studies for the given SNP, such that it has correlation matrix $\boldsymbol{P}_{\mathbf{G}}$, as follows:

$$\boldsymbol{\beta}_k = \begin{pmatrix} \beta_{1k} \\ \vdots \\ \beta_{Ck} \end{pmatrix} = \operatorname{diag}\left(\sigma_{\beta_1}, \ldots \sigma_{\beta_C}\right) \mathbf{\Gamma}_{\mathbf{G}} \boldsymbol{\eta}_k,$$

where diag() is a diagonal matrix with specified elements as diagonal entries, and

$$\sigma_{\beta_j} = \sqrt{\frac{h_j^2 \sigma_{\mathbf{y}_j}^2}{M}},$$

with h_j^2 (resp. $\sigma_{\mathbf{y}_j}^2$) denoting the SNP heritability (phenotypic variance) in study j. Under this design of study-specific SNP effects, we attain a CGR structure in line with $\boldsymbol{P}_{\mathbf{G}}$ and the desired study-specific SNP heritabilities.

Using this approach for constructing correlated SNP effects, we can write the effect of SNP k in study j (i.e.,

β_{jk}) as a linear combination of the independent underlying $\mathcal{N}(0,1)$ distributed random variables. That is,

$$\beta_{jk} = \sigma_{\beta_j} \sum_{i=1}^{j} \gamma_{ji}\eta_{ik}, \tag{10}$$

where γ_{ji} denotes element in row j column i of $\mathbf{\Gamma}$ and η_{ik} the i-th element of $\boldsymbol{\eta}_k$. Given our scaling of SNPs, the R^2 of each causal SNP in study j is given by $\sigma^2_{\beta_j}$, regardless of the allele frequency of the SNP of interest (Assumption 5).

We can now write the GWAS Z statistic for a given SNP in a given study, as a linear combination of independent random variables. For SNP k in the set of P non-causal SNPs, denoted by $\mathcal{P}$ (such that $\mathcal{M} \cap \mathcal{P} = \varnothing$), we have for all studies j that $\beta_{jk} = 0$. By substituting β in Eq. 9 according to Eq. 10 for causal SNPs and the preceding equality for non-causal SNPs, we obtain the following expression for the Z statistic of SNP k in study j:

$$Z_{jk} = \begin{cases} v_{jk} + \sqrt{N_j}\frac{\sigma_{\beta_j}}{\sigma_{\boldsymbol{\epsilon}_j}} \sum_{i=1}^{j} \gamma_{ji}\eta_{ik} & \text{for} \quad k \in \mathcal{M}, \text{ and} \\ v_{jk} & \text{for} \quad k \in \mathcal{P}. \end{cases} \tag{11}$$

Distribution meta-analysis Z statistic Here, we derive the distribution of the meta-analysis Z statistic and reduce the number of input parameters by appropriate substitutions. Finally, for intuition, we present the distribution of Z statistics from a meta-analysis of GWAS results from two studies.

For any SNP k in the set $\mathcal{S} = \mathcal{M} \cup \mathcal{P}$ consisting of S $= M + P$ causal and non-causal SNPs, we use the sample-size-weighted meta-analysis Z statistic [6], defined as follows:

$$Z_k = \sum_{j=1}^{C} \frac{\sqrt{N_j}}{\sqrt{N_T}} Z_{jk}, \tag{12}$$

where $N_T = N_1 + \ldots + N_C$ denotes the total sample size. Plugging Eq. 11 for $k \in \mathcal{M}$ into Eq. 12, yields an expression for the meta-analysis Z statistic in terms of independent random variables. That is,

$$Z_k = \begin{cases} \sum_{j=1}^{C} \frac{\sqrt{N_j}}{\sqrt{N_T}} v_{jk} + \sum_{j=1}^{C} \sum_{i=1}^{j} \frac{N_j}{\sqrt{N_T}} \frac{\sigma_{\beta_j}}{\sigma_{\boldsymbol{\epsilon}_j}} \gamma_{ji}\eta_{ik} & \text{for} \quad k \in \mathcal{M}, \text{ and} \\ \sum_{j=1}^{C} \frac{\sqrt{N_j}}{\sqrt{N_T}} v_{jk} & \text{for} \quad k \in \mathcal{P}. \end{cases} \tag{13}$$

As the v_{jk} terms in the preceding expression are independent standard-normal draws, it follows that

$$v_k = \sum_{j=1}^{C} \frac{\sqrt{N_j}}{\sqrt{N_T}} v_{jk} \sim \mathcal{N}(0,1).$$

In Eq. 13 we have a double sum over random variables. However, by changing the order of summation, this

double sum can be rewritten as follows:

$$\sum_{j=1}^{C}\sum_{i=1}^{j}\frac{N_j}{\sqrt{N_T}}\frac{\sigma_{\beta_j}}{\sigma_{\boldsymbol{\varepsilon}_j}}\gamma_{ji}\eta_{ik}=\sum_{i=1}^{C}\eta_{ik}\sum_{j=i}^{C}\frac{N_j}{\sqrt{N_T}}\frac{\sigma_{\beta_j}}{\sigma_{\boldsymbol{\varepsilon}_j}}\gamma_{ji}.$$

Therefore, we can rewrite Eq. 13 as follows:

$$Z_k=\begin{cases} v_k+\sum_{i=1}^{C}\eta_{ik}\sum_{j=i}^{C}\frac{N_j}{\sqrt{N_T}}\frac{\sigma_{\beta_j}}{\sigma_{\boldsymbol{\varepsilon}_j}}\gamma_{ji} & \text{for} \quad k\in\mathcal{M}, \text{ and}\\ v_k & \text{for} \quad k\in\mathcal{P}, \end{cases} \tag{14}$$

where the inner sum yields the weight for the random variable of interest.

Exploiting the fact that η_{ik} and v_k are independent standard-normal draws, the variance of the sum of terms is equal to the sum of the variance of the respective terms. Hence, we have that

$$Z_k\sim\begin{cases} \mathcal{N}(0,1+d) & \text{for} \quad k\in\mathcal{M}, \text{ and}\\ \mathcal{N}(0,1) & \text{for} \quad k\in\mathcal{P}, \end{cases}$$

where

$$d=\sum_{i=1}^{C}\left(\sum_{j=i}^{C}\frac{N_j}{\sqrt{N_T}}\frac{\sigma_{\beta_j}}{\sigma_{\boldsymbol{\varepsilon}_j}}\gamma_{ji}\right)^2 \tag{15}$$

$$=\frac{1}{N_T}\sum_{i=1}^{C}\left(\sum_{j=i}^{C}N_j\frac{\sigma_{\beta_j}}{\sigma_{\boldsymbol{\varepsilon}_j}}\gamma_{ji}\right)^2 \tag{16}$$

The quantity d we refer to as the 'power parameter'. Since this parameter is a sum of squares, it is non-negative. The greater the power parameter is, the higher the statistical power of the meta-analysis of GWAS results is. Note that in case $\sigma_{\beta_j}=0$ for all j (i.e., the trait is not heritable in any study), that $d=0$, and hence the meta-analysis Z statistic reverts to a standard-normal test statistic, which matches the distribution under the null. However, as σ_{β_j} increases, d becomes larger, yielding a meta-analysis with higher statistical power.

Given SNP-based heritability, phenotypic variation, and the number of causal variants, we have that the effect size per causal SNP in a study is given by $\sigma^2_{\beta_j}=\frac{h^2_j\sigma^2_{\mathbf{y}_j}}{M}$, and the residual variance, absorbing the variance due to the omitted $M-1$ SNPs (Assumption 1), is given by $\sigma^2_{\boldsymbol{\varepsilon}_j}=\sigma^2_{\mathbf{y}_j}-\sigma^2_{\beta_j}$. Using these expressions, we can write the ratio

of σ_{β_j} and σ_{ε_j}, appearing in Eq. 16, as a function of only heritability and the number of causal SNPs. That is,

$$\frac{\sigma_{\beta_j}}{\sigma_{\varepsilon_j}} = \sqrt{\frac{\frac{h_j^2 \sigma_{\mathbf{y}_j}^2}{M}}{\sigma_{\mathbf{y}_j}^2 - \frac{h_j^2 \sigma_{\mathbf{y}_j}^2}{M}}} \tag{17}$$

$$= \sqrt{\frac{h_j^2}{M - h_j^2}}. \tag{18}$$

Plugging the last expression into Eq. 16 yields

$$d = \frac{1}{N_T} \sum_{i=1}^{C} \left(\sum_{j=i}^{C} N_j \sqrt{\frac{h_j^2}{M - h_j^2}} \gamma_{ji} \right)^2 \tag{19}$$

This expression for the power parameter shows that it is not affected by scaling due to phenotypic variance; the parameter is only affected by the cross-study genetic correlation matrix, the SNP-based heritability per study, and the sample size per study.

In case the number of studies is two, with sample size N in Study 1 and N in Study 2, SNP heritability h_{SNP}^2, and a genetic correlation $\rho_{\mathbf{G}}$ between the two studies, we have that the meta-analysis Z statistic, of a trait-affecting SNP k, is normally distributed with mean zero and

$$\text{Var}\left(Z_{k,C=2}\right) = 1 + \frac{h_{\text{SNP}}^2}{M - h_{\text{SNP}}^2} N \left(1 + \rho_{\mathbf{G}}\right).$$

Bearing in mind that the number of causal SNPs $M \gg 1$ under a highly polygenic model, while $h_{\text{SNP}}^2 \in [0,1]$, we have that under high polygenicity $M - h_{\text{SNP}}^2 \approx M$. Hence, the variance of Z_k can be approximated by

$$\text{Var}\left(Z_{k,C=2,\text{high polygenicity}}\right) \approx 1 + \frac{h_{\text{SNP}}^2}{M} N \left(1 + \rho_{\mathbf{G}}\right).$$

In the scenario where the cross-study genetic correlations equals one, we have that $\text{Var}\left(Z_k\right) \approx 1 + \frac{h_{\text{SNP}}^2}{M} N_T$ for a trait-affecting haplotype block and $\text{Var}\left(Z_k\right) = 1$ for a non-causal haplotype block, where $N_T = 2N$. These expressions are equivalent to the expected value of the squared Z statistics from the linear regression analysis reported in Section 4.2 of the Supplementary Note to [3], as well as the first equation in [5] when assuming that confounding biases and linkage disequilibrium are absent.

Adding genetically uncorrelated studies to the meta-analysis Here, we consider what happens to statistical power of a meta-analysis of GWAS results from several sets of studies, with genetic correlations between the studies within each set, but with no genetic correlation between the different sets. We first consider a scenario with one set

consisting of $C-1$ studies and one other set consisting of only one study. We then generalize to a setting with multiple sets, each set containing at least one study. We show that the power parameter for a meta-analysis of several sets of studies with no genetic correlations between sets, can be written as a sample-size weighted sum of the power parameters within the respective sets.

In case one has $C-1$ studies with associated CGR matrix, the associated Cholesky decomposition denoted by $\boldsymbol{\Gamma}_{(C)}$, and an additional study indexed by C, which is genetically uncorrelated to the $C-1$ other studies, then the $C \times C$ Cholesky decomposition of the full CGR matrix is given by

$$\boldsymbol{\Gamma}_{\mathbf{G}} = \begin{pmatrix} \boldsymbol{\Gamma}_{(C)} & \mathbf{0} \\ \mathbf{0}^{\top} & 1 \end{pmatrix},$$

where $\mathbf{0}$ denotes a column vector of zeros.

Now, the quantity d in Eq. 19 can be decomposed as follows.

$$d = \frac{1}{N_T} \sum_{i=1}^{C-1} \left(\sum_{j=i}^{C-1} N_j \sqrt{\frac{h_j^2}{M-h_j^2}} \gamma_{ji} \right)^2 + \frac{1}{N_T} \left(N_C \sqrt{\frac{h_C^2}{M-h_C^2}} \right)^2 \tag{20}$$

$$= \frac{N_{(C)}}{N_T} \frac{1}{N_{(C)}} \sum_{i=1}^{C-1} \left(\sum_{j=i}^{C-1} N_j \sqrt{\frac{h_j^2}{M-h_j^2}} \gamma_{ji} \right)^2 + \frac{N_C}{N_T} \frac{1}{N_C} \left(N_C \sqrt{\frac{h_C^2}{M-h_C^2}} \right)^2 \tag{21}$$

$$= \frac{N_{(C)}}{N_T} d_{(C)} + \frac{N_C}{N_T} d_C, \tag{22}$$

where d_C denotes the power parameter in Eq. 19 had only study C (with sample-size N_C) been considered, and $d_{(C)}$ the power parameter in Eq. 19 had only the first $C-1$ studies (with total corresponding sample-size $N_{(C)}$) been considered. Hence, the power parameter in this scenario is the sample-size-weighted average of the power parameter of the first $C-1$ studies jointly and the power parameter of the last study.

Eq. 22 can be generalized, to reflect a situation where there are P disjoint sets of studies, denoted by $\mathcal{C}_1, \ldots, \mathcal{C}_P$, with genetic correlation within each set, but no genetic correlation between the sets. In this scenario, the power parameter d in Eq. 19 for a joint meta-analysis of all sets is given by

$$d_{\mathcal{C}_1 \cup \mathcal{C}_2 \cup \ldots \cup \mathcal{C}_P} = \sum_{p=1}^{P} \frac{N_{\mathcal{C}_p}}{N_T} d_{\mathcal{C}_p}, \tag{23}$$

where $N_{\mathcal{C}_p}$ denotes the total sample size in study-set $\mathcal{C}_p$ and $d_{\mathcal{C}_p}$ the power parameter in Eq. 19 for the meta-analysis of all studies in set $\mathcal{C}_p$, and N_T the total sample size when aggregating over all study sets. This equation states that power parameter for a meta-analysis of several sets of studies with CGR within each set, but no CGR between sets, is a weighted average of the power parameters in the underlying sets.

Since the statistical power is a monotonically increasing function of the power parameter d, Eq. 23 leads to two corollaries under CGR equal to zero between sets of studies, namely that

$$\beta_{\mathcal{C}_1 \cup \mathcal{C}_2 \cup \ldots \cup \mathcal{C}_P} \leq \max \left\{ \beta_{\mathcal{C}_p} \right\}_{p=1,\ldots,P} \text{ and} \tag{24}$$

$$\beta_{\mathcal{C}_1 \cup \mathcal{C}_2 \cup \ldots \cup \mathcal{C}_P} \geq \min \left\{ \beta_{\mathcal{C}_p} \right\}_{p=1,\ldots,P} , \tag{25}$$

where $\beta_{\mathcal{A}}$ denotes the power in set of studies $\mathcal{A}$.

The implication of Eq. 23 is simple yet powerful; when several sets of studies with genetic correlation within each set, but no genetic correlation between sets, are considered for meta-analysis, one should not meta-analyze sets $\mathcal{C}_1, \ldots \mathcal{C}_P$ jointly, but rather meta-analyze only the set of studies which has the largest power parameter according to Eq. 19.

Only when $d_{\mathcal{C}_1 \cup \mathcal{C}_2 \cup \ldots \cup \mathcal{C}_P} > \max \{d_{\mathcal{C}_1}, \ldots, d_{\mathcal{C}_P}\}$, does the meta-analysis of all sets jointly have higher statistical power than a meta-analysis based on only one set of studies.

References

1. Yang J, Lee SH, Goddard ME, Visscher PM. GCTA: a tool for genome-wide complex trait analysis. Am J Hum Genet. 2011;88:76–82.

2. Lee SH, Yang J, Goddard ME, Visscher PM, Wray NR. Estimation of pleiotropy between complex diseases using single-nucleotide polymorphism-derived genomic relationships and restricted maximum likelihood. Bioinformatics. 2012;28:2540–2542.

3. Yang J, Zaitlen NA, Goddard ME, Visscher PM, Price AL. Advantages and pitfalls in the application of mixed-model association methods. Nat Genet. 2014;46:100–106.

4. Yang J, Weedon MN, Purcell SM, Lettre G, Estrada K, Willer CJ, et al. Genomic inflation factors under polygenic inheritance. Eur J Hum Genet. 2011;19:807–812.

5. Bulik-Sullivan BK, Loh PR, Finucane HK, Ripke S, Yang J, Patterson N, et al. LD Score regression distinguishes confounding from polygenicity in genome-wide association studies. Nat Genet. 2015;47:291–295.

6. Willer CJ, Li Y, Abecasis GR. METAL: fast and efficient meta-analysis of genomewide association scans. Bioinformatics. 2010;26:2190–2191.
