# Supplementary material for: Meta-GWAS Accuracy and Power (MetaGAP) Calculator Shows that Hiding Heritability Is Partially Due to Imperfect Genetic Correlations across Studies
Source: PLoS Genet. 2017 Jan 17;13(1):e1006495. doi: 10.1371/journal.pgen.1006495 (PMC5240919; doi:10.1371/journal.pgen.1006495)
Supplement: S2 Derivations — (PDF) [file pgen.1006495.s002.pdf]

S2 Derivations. Predictive accuracy of a polygenic score based on SNP-effect estimates from a meta-analysis of GWAS results.

In this supporting information section, we extend the theoretical framework for meta-analytic power discussed in S1 Derivations. The derivations in this section are based on the same assumptions as in S1 Derivations. We consider the predictive accuracy of the polygenic score (PGS) including all S independent SNPs, with SNP-weights based on the meta-analysis results from the set of C studies, in a hold-out sample indexed as 'study' $C + 1$. In this hold-out sample, we focus exclusively on the theoretical R^2 of the PGS; instead of considering multiple draws from the stochastic processes underlying the genotypes and treating these as fixed explanatory variables, we treat the phenotype, the PGS, and the underlying genotypes as random variables, and use probability theory to derive R^2. The hold-out sample is also allowed a study-specific SNP-based heritability, h^2_{C+1}, and genetic-correlations with the other C studies (thus extending both the CGR matrix and its Cholesky decomposition to $(C + 1)\times(C + 1)$ matrices).

First, we write the phenotype in hold-out sample as a function of noise and the independent genetic factors discussed in the preceding section. Second, we derive an expression for the PGS as a function of the genetic factors. Third, using this representation we derive the theoretical covariance between the PGS and the phenotype. Fourth, using the theoretical variances and covariance, we obtain an expression for the theoretical R^2.

Polygenic model Here, we derive an expression for the phenotype in the hold-out study as a function of independent genetic factors and an expression for the phenotypic variance.

Aggregating across causal SNP set $\mathcal{M}$ and the noise, the phenotype in study $C + 1$ can be written as follows:

$$Y_{C+1} = \sum_{k\in\mathcal{M}} X_{C+1,k}\beta_{C+1,k} + \varepsilon_{C+1},$$

where, analogous to Eq. 10 in S1 Derivations,

$$\beta_{C+1,k} = \sigma_{\beta_{C+1,k}} \sum_{i=1}^{C+1} \gamma_{C+1,i}\eta_{ik},$$

where η_{ik} now indicates the i-th element of the now $(C + 1)$-dimensional vector of independent normal draws, $\boldsymbol{\eta}_k$, and where $\gamma_{C+1,i}$ describes an element of the Cholesky decomposition $\mathbf{\Gamma_G}$ of the $(C + 1)\times(C + 1)$ cross-study genetic correlation matrix, incorporating the hold-out sample. Hence, the phenotype can be written as

$$Y_{C+1} = \varepsilon_{C+1} + \sum_{k\in\mathcal{M}} \left(X_{C+1,k}\sigma_{\beta_{C+1,k}} \sum_{i=1}^{C+1} \gamma_{C+1,i}\eta_{ik} \right).$$

Analogous to the scaling of SNPs in S1 Derivations here, with genotypes treated as random variables, we assume

$$\mathbb{E}\left[X_{C+1,k}\right] = 0 \text{ and } \mathrm{Var}\left(X_{C+1,k}\right) = 1, \text{ for } k \in \mathcal{S}, \text{ and}$$
$$\mathrm{Cov}\left(X_{C+1,k}, X_{C+1,l}\right) = 0 \text{ for } k \neq l.$$

Consequently, the phenotypic variance in the hold-out sample is given by

$$\mathrm{Var}\left(Y_{C+1}\right) = M\sigma^2_{\beta_{C+1}} + \sigma^2_{\varepsilon_{C+1}}. \tag{1}$$

Polygenic score Here, we derive an expression for the PGS as a function of independent genetic factors, an expression for the PGS variance, and its covariance with the phenotype in the hold-out sample.

Since each SNP in each study in the meta-analysis has been scaled such that its dot product equals the sample size of that study, by analogy of the standard error of the SNP effect estimate in a single study, the standard-error of the meta-analytic effect estimate $\widehat{\beta}_{meta}$ for study $C + 1$ can be approximated by

$$\text{s.d.}\left(\widehat{\beta}_{meta}\right) \propto \frac{1}{\sqrt{N_T}} \propto 1,$$

where N_T denotes the total sample size of the meta analysis.

Hence, the meta-analytic effect estimate is proportional to the meta-analysis Z statistic. Since any scalar multiple of the PGS will not affect its R^2 with respect to the phenotype, the Z statistics of the meta-analysis can be applied as SNP weights directly. Therefore, the PGS in the hold-out sample, including all SNPs, is given by

$$\widehat{Y}_{C+1} = \sum_{k\in\mathcal{S}} X_{C+1,k} Z_k. \tag{2}$$

Plugging the expression for Z_k from Eq. 14 in S1 Derivations into Eq. 2, and substitution of terms by means of the square root of Eq. 18 in S1 Derivations, the PGS is given by

$$\widehat{Y}_{C+1} = \left(\sum_{k\in\mathcal{S}} X_{C+1,k} v_k\right) + \left(\sum_{k\in\mathcal{M}} X_{C+1,k} \sum_{i=1}^{C} \eta_{ik} \sum_{j=i}^{C} \frac{N_j}{\sqrt{N_T}} \sqrt{\frac{h_j^2}{M - h_j^2}} \gamma_{ji}\right).$$

Exploiting the fact that η_{ik}, v_k, and $X_{C+1,k}$ are all independent random variables, with mean zero and variance one, we find that the variance of the PGS is given by

$$\mathrm{Var}\left(\widehat{Y}_{C+1}\right) = S + M \sum_{i=1}^{C} \left(\sum_{j=i}^{C} \frac{N_j}{\sqrt{N_T}} \sqrt{\frac{h_j^2}{M - h_j^2}} \gamma_{ji}\right)^2. \tag{3}$$

Again exploiting independence, zero mean, and unit variance of the respective terms, the covariance between the PGS and the phenotype is given by

$$\operatorname{Cov}\left(Y_{C+1}, \widehat{Y}_{C+1}\right) = \mathbb{E}\left[Y_{C+1}\widehat{Y}_{C+1}\right] \tag{4}$$

$$= \begin{matrix} \mathbb{E}\left[\left(\sum_{k\in\mathcal{M}} X_{C+1,k}\sigma_{\beta_{C+1,k}} \sum_{i=1}^{C+1} \gamma_{C+1,i}\eta_{ik}\right)\dots\right. \\ \left.\cdot\left(\sum_{k\in\mathcal{M}} X_{C+1,k} \sum_{i=1}^{C} \eta_{ik} \sum_{j=i}^{C} \frac{N_j}{\sqrt{N_T}}\sqrt{\frac{h_j^2}{M-h_j^2}}\gamma_{ji}\right)\right]\end{matrix} \tag{5}$$

$$= \mathbb{E}\left[\left(\sum_{k\in\mathcal{M}} X_{C+1,k}^2\sigma_{\beta_{C+1,k}}\left(\sum_{i=1}^{C}\gamma_{C+1,i}\eta_{ik}^2\sum_{j=i}^{C}\frac{N_j}{\sqrt{N_T}}\sqrt{\frac{h_j^2}{M-h_j^2}}\gamma_{ji}\right)\right)\right] \tag{6}$$

$$= \sigma_{\beta_{C+1,k}} M\left(\sum_{i=1}^{C}\sum_{j=i}^{C}\frac{N_j}{\sqrt{N_T}}\sqrt{\frac{h_j^2}{M-h_j^2}}\gamma_{C+1,i}\gamma_{ji}\right). \tag{7}$$

Theoretical R^2 Here, we derive the theoretical R^2 between the PGS and the phenotype in a hold-out study. For intuition, we present the theoretical R^2 for a scenario with one study for discovery and one study as hold-out sample.

By combining Eq. 1, 3, and 7, the R^2, defined as the squared correlation of the outcome and the PGS in the hold-out sample, is now given by

$$R^2\left(Y_{C+1}, \widehat{Y}_{C+1}\right) = \frac{\left(\operatorname{Cov}\left(Y_{C+1}, \widehat{Y}_{C+1}\right)\right)^2}{\operatorname{Var}\left(Y_{C+1}\right)\operatorname{Var}\left(\widehat{Y}_{C+1}\right)}$$

$$= \frac{\sigma_{\beta_{C+1,k}}^2 M^2\left(\sum_{i=1}^{C}\sum_{j=i}^{C}\frac{N_j}{\sqrt{N_T}}\sqrt{\frac{h_j^2}{M-h_j^2}}\gamma_{C+1,i}\gamma_{ji}\right)^2}{\left(M\sigma_{\beta_{C+1}}^2+\sigma_{\varepsilon_{C+1}}^2\right)\left(S+M\sum_{i=1}^{C}\left(\sum_{j=i}^{C}\frac{N_j}{\sqrt{N_T}}\sqrt{\frac{h_j^2}{M-h_j^2}}\gamma_{ji}\right)^2\right)}.$$

This expression can be simplified as follows:

$$R^2\left(Y_{C+1}, \widehat{Y}_{C+1}\right) = h_{C+1}^2\frac{n}{\frac{S}{M}+d}, \tag{8}$$

where d is the meta-analysis power parameter given in Eq. 19 in S1 Derivations and numerator n is given by

$$n = \frac{1}{N_T}\left(\sum_{i=1}^{C}\sum_{j=i}^{C}N_j\sqrt{\frac{h_j^2}{M-h_j^2}}\gamma_{C+1,i}\gamma_{ji}\right)^2,$$

where N is the total sample size in the meta-analysis.

The expression for R^2 in Eq. 8 is such that, in addition to the parameters needed for the power calculation, one

only needs the genetic correlation between the hold-out sample and the meta-analysis samples and the heritability in the hold-out sample.

In case there is only one discovery study (i.e., $C = 1$) with sample size N, and with a genetic correlation $\rho_{\mathbf{G}}$ between the hold-out and discovery sample, we have that

$$R^2_{C=1} = h_2^2 \rho_{\mathbf{G}}^2 \frac{\frac{N h_1^2}{M - h_1^2}}{\frac{S}{M} + \frac{N h_1^2}{M - h_1^2}}.$$

As in S1 Derivations, we have that under high polygenicity $M - h_1^2 \approx M$. Therefore, an easy approximation of R^2 in this scenario is given by

$$R^2_{C=1,\text{high polygenicity}} \approx h_2^2 \rho_{\mathbf{G}}^2 \frac{h_1^2}{\frac{S}{N} + h_1^2}.$$

When $\rho_{\mathbf{G}}^2 = 1$, S=M, and $h_1^2 = h_2^2$, we obtain a known expression for PGS R^2 in terms of sample size, heritability, and the number of SNPs [1]. In case $\rho_{\mathbf{G}}^2 = 1$ and we consider the R^2 between the PGS and genetic value (i.e., the genetic component of the phenotype), both $\rho_{\mathbf{G}}^2$ and h_2^2 can be ignored, thereby making the last expression equivalent to the first equation in [2].

References

1. Dudbridge F. Power and predictive accuracy of polygenic risk scores. PLOS Genet. 2013;9:e1003348.
2. Daetwyler HD, Villanueva B, Woolliams JA. Accuracy of predicting the genetic risk of disease using a genome-wide approach. PLOS ONE. 2008;3:e3395.
