# Supplementary material for: Meta-GWAS Accuracy and Power (MetaGAP) Calculator Shows that Hiding Heritability Is Partially Due to Imperfect Genetic Correlations across Studies
Source: PLoS Genet. 2017 Jan 17;13(1):e1006495. doi: 10.1371/journal.pgen.1006495 (PMC5240919; doi:10.1371/journal.pgen.1006495)
Supplement: S1 Note — (PDF) [file pgen.1006495.s003.pdf]

S1 Note. Genetic correlations.

Consider, without loss of generality, a model for two phenotypes, Y_1 and Y_2. Similar to S2 Derivations, we treat phenotypes, genotypes, and SNP effects as random variables. In line with Assumption 5 in S1 Derivations, let each causal variant, for the phenotype of interest, have the same R^2 with respect to that phenotype.

We can write the data-generating processes of the respective phenotypes as

$$Y_1 = \sum_{k \in \mathcal{M}_1} X_{k,1}\beta_{k,1} + \varepsilon_1 \text{ and}$$
$$Y_2 = \sum_{p \in \mathcal{M}_2} X_{p,2}\beta_{p,2} + \varepsilon_2,$$

where $\mathcal{M}_1$ (resp. $\mathcal{M}_2$) denotes the set of causal SNPs for Y_1 (Y_2) and where $\beta_{k,1}$ (resp. $\beta_{p,2}$) the effect of $X_{k,1}$ ($X_{p,2}$), that is, standardized SNP k (p), on phenotype 1 (2).

The genetic correlation at the genome-wide level can now be conceptualized as the correlation in the true genetic value for both phenotypes. That is

$$\begin{aligned}\rho_{\mathbf{G}} &= \operatorname{Corr}\left(\sum_{k \in \mathcal{M}_1} X_{k,1}\beta_{k,1}, \sum_{p \in \mathcal{M}_2} X_{p,2}\beta_{p,2}\right) \\ &= \frac{\operatorname{Cov}\left(\sum_{k \in \mathcal{M}_1} X_{k,1}\beta_{k,1}, \sum_{p \in \mathcal{M}_2} X_{p,2}\beta_{p,2}\right)}{\sqrt{\operatorname{Var}\left(\sum_{k \in \mathcal{M}_1} X_{k,1}\beta_{k,1}\right) \operatorname{Var}\left(\sum_{p \in \mathcal{M}_2} X_{p,2}\beta_{p,2}\right)}}\end{aligned}$$

Assuming independent haplotype blocks with independent effects (Assumption 4 in S1 Derivations), where the effects have mean zero, this expression for the genetic correlation at the genome-wide level can be rewritten as

$$\begin{aligned}\rho_{\mathbf{G}} &= \frac{\sum_{k \in \{\mathcal{M}_1 \cap \mathcal{M}_2\}} \mathbb{E}\left[\beta_{k,1}\beta_{k,2}\right]}{\sqrt{|\mathcal{M}_1|\sigma^2_{\beta_1}|\mathcal{M}_2|\sigma^2_{\beta_2}}} \\ &= \frac{|\mathcal{M}_1 \cap \mathcal{M}_2|}{\sqrt{|\mathcal{M}_1||\mathcal{M}_2|}} \frac{\sigma_{\beta_{1,2}}}{\sqrt{\sigma^2_{\beta_1}\sigma^2_{\beta_2}}},\end{aligned}$$

where $|\mathcal{A}|$ denotes the number of elements in set $\mathcal{A}$.

Hence, the genetic correlation at the genome-wide level can be written as the product of overlap in causal loci between the two traits and the cross-trait correlation of the effects of these overlapping loci. That is,

$$\rho_{\mathbf{G}} = \frac{|\mathcal{M}_1 \cap \mathcal{M}_2|}{\sqrt{|\mathcal{M}_1||\mathcal{M}_2|}}\rho_\beta. \tag{1}$$

Eq. 1 is a generalization of the 'common-elements formula' [1], describing a correlation as a function of the number

of overlapping elements and unique elements.

In particular, when $|\mathcal{M}_1| = |\mathcal{M}_2|$, we have that

$$\rho_{\mathbf{G}} = \frac{O}{O+D}\rho_{\beta},$$

where O denotes the number of overlapping causal loci and D the number of idiosyncratic causal loci per trait.

We assume throughout the paper that all causal loci are shared across traits and studies (Assumption 3 in S1 Derivations). That is,

$$\frac{|\mathcal{M}_1 \cap \mathcal{M}_2|}{\sqrt{|\mathcal{M}_1||\mathcal{M}_2|}} = 1,$$

and that, consequently, the genetic correlation at the genome-wide level is equal to the correlation in the effects of overlapping causal SNPs. That is,

$$\rho_{\mathbf{G}} = \rho_{\beta}.$$

As we show in S1 Simulations, the theoretical predictions of GWAS power and predictive accuracy obtained under this assumption are quite accurate, even when an imperfect genetic correlation at the genome-wide level is shaped primarily by lack of overlap in causal loci, rather than a poor correlation in the effects of overlapping loci.

References

1. Jensen AR. Note on why genetic correlations are not squared. Psychol Bull. 1971;75:223–224.
