# Supplementary material for: Meta-GWAS Accuracy and Power (MetaGAP) Calculator Shows that Hiding Heritability Is Partially Due to Imperfect Genetic Correlations across Studies
Source: PLoS Genet. 2017 Jan 17;13(1):e1006495. doi: 10.1371/journal.pgen.1006495 (PMC5240919; doi:10.1371/journal.pgen.1006495)
Supplement: S1 Simulations — (PDF) [file pgen.1006495.s004.pdf]

S1 Simulations. Assessment of the accuracy of the MetaGAP calculator.

Using five simulation studies, we assess the accuracy of the MetaGAP calculator, which is based on the expressions for GWAS power and PGS R^2 derived in S1 Derivations and S2 Derivations. Since the calculator is based on specific assumptions regarding the data-generating process, an important question is whether the calculator still provides accurate predictions of power and R^2 when the underlying assumptions are violated.

Hence, each simulation study has a different underlying data-generating process. The first study, Simulation 1, assumes that rare variants have larger effects than common variants to such an extent that each causal SNP, regardless of allele frequency, is expected to have the same R^2 with respect to the phenotype (Assumption 5 in S1 Derivations). This simulation is entirely in line with the assumptions underlying the MetaGAP calculator. In the second study, Simulation 2, common variants have effects of the same magnitude as rare variants (leading a common causal variant to explain a larger proportion of the phenotypic variation than a rare causal variant). The third study, Simulation 3, also allows for differential R^2 between SNPs and, in addition, does not assume that SNP allele frequencies are uniformly distributed. Instead, the third study assumes that there are more variants in the lower minor allele frequency bins than in the higher minor allele frequency bins. In addition to the deviations from assumptions made in Simulations 2 and 3, Simulation 4 allows allele frequencies to be completely independent across studies. Finally, in Simulation 5, we go back to a data-generating process in line with the assumptions underlying the MetaGAP calculator, with one important difference; in Simulation 5, the genetic correlation as inferred at the genome-wide level is not only shaped by the correlation of SNP effects, but also by the degree of overlap of causal loci across studies. Thereby, Simulation 5 violates the assumption discussed in S1 Note, that the estimated CGR is shaped only by imperfect correlations of SNP effects across studies.

For each simulation study there are 100 independent runs. In each run data is simulated for $C = 3$ distinct samples for discovery as well as a fourth sample used as hold-out sample for prediction. The sample sizes of the respective studies are given by $N_1 = 20{,}000$, $N_2 = 15{,}000$, $N_3 = 10{,}000$, and $N_4 = 1{,}000$, where N_4 denotes the sample size of the hold-out sample. For Simulations 1–4, an 11×11 grid of equispaced values of $h^2_{\text{SNP}} \in [0, 1]$ and $\rho_\beta \in [0, 1]$ is considered. Similarly, for Simulation 5, an 11×11 grid of equispaced values of $s \in [0, 1]$ and $\rho_\beta \in [0, 1]$ is considered. Here, s denotes the fraction of causal SNPs that overlaps across studies and ρ_β the cross-study correlation of the effects of SNPs that are overlapping. In Simulations 1–4 we have that $s = 1$ and in Simulation 5 we have that $h^2_{\text{SNP}} = 0.5$. In all simulations there are $S = 100{,}000$ independent SNPs of which $M = 1{,}000$ have a causal influence. Moreover, when computing theoretical power and predictive accuracy, in line with S1 Note, we use $\rho_{\mathbf{G}} = s \cdot \rho_{\mathbf{G}}$ as value of the input parameter CGR. A detailed description of the data-generating process in each simulation study can be found in Table A1.

For every run, data is simulated in accordance with the underlying data-generating process. Next, a GWAS

Table A1. Design of Simulations 1–5. Design settings identical to a preceding simulation study are denoted by 'idem', followed by the number of the first preceding simulation study with the same setting.

Data-generating process*	Notation	Simulation 1	Simulation 2	Simulation 3	Simulation 4	Simulation 5
# studies for meta-analysis	$C =$	3	idem 1	idem 1	idem 1	idem 1
Index prediction sample	$C + 1 =$	4	idem 1	idem 1	idem 1	idem 1
Sample size per study	$\{N_1, N_2, N_3, N_4\} =$	{20k; 15k; 10k; 1k}	idem 1	idem 1	idem 1	idem 1
Effective # SNPs	$\lvert\mathcal{S}\rvert = S =$	100k	idem 1	idem 1	idem 1	idem 1
Effective # causal SNPs, study j**	$\lvert\mathcal{M}_j\rvert = M =$	1k	idem 1	idem 1	idem 1	idem 1
Overlap causal SNPs, studies $\{j, h\}$***	$s_{jh} = \frac{\lvert\mathcal{M}_j \cap \mathcal{M}_h\rvert}{M} \in$	1	idem 1	idem 1	idem 1	$\{0, 0.1, \ldots, 1\}$
SNP-based heritability***	$h^2_{\text{SNP}} \in$	{0%, 10%, ..., 100%}	idem 1	idem 1	idem 1	50%
CGR of overlapping SNPs***	$\rho_\beta \in$	$\{0, 0.1, \ldots, 1\}$	idem 1	idem 1	idem 1	idem 1
Allele frequency SNP $k \in \mathcal{S}$, study j	$f_{jk} \sim$	$\mathcal{U}(0.05, 0.95)$	idem 1	*Beta*(0.35,0.35)****	idem 3	idem 1
Frequency SNP k, studies $j \neq h$	$=$ or $\perp\!\!\!\perp$*****	$=$	idem 1	idem 1	$\perp\!\!\!\perp$	idem 1
Genotype k, individual i, study j	$G_{jik} \sim$	$Binom(2, f_{jk})$	idem 1	idem 1	idem 1	idem 1
Effect SNP $k \notin \mathcal{M}, j$	$\beta_{jk} =$	0	idem 1	idem 1	idem 1	idem 1
Effect SNP $k \in \mathcal{M}, j$	$\beta_{jk} \sim$	$\mathcal{N}(0, 1)$	idem 1	idem 1	idem 1	idem 1
Correlation SNP effect k, $j \neq h$	$\text{corr}(\beta_{jk}, \beta_{hk}) =$	ρ_β	idem 1	idem 1	idem 1	idem 1
Residual i, j	$\varepsilon_{ji} \sim$	$\mathcal{N}\left(0, 1 - h^2_{\text{SNP}}\right)$	idem 1	idem 1	idem 1	idem 1
Genetic value i, j	$GV_{ji} =$	$\sum_{k \in \mathcal{M}_j} \frac{G_{jik} - 2f_{jk}}{\sqrt{2f_{jk}(1-f_{jk})}} \beta_{jk}$	$\sum_{k \in \mathcal{M}_j} (G_{jik} - 2f_{jk})\beta_{jk}$	idem 2	idem 2	idem 1
GV coefficient j	$c_j =$	$\sqrt{h^2_{\text{SNP}}/M}$	$\sqrt{\frac{h^2_{\text{SNP}}}{\frac{1}{N_j}\sum_{i=1}^{N_j} GV^2_{ji}}}$	idem 2	idem 2	idem 1
Phenotype i,j	$Y_{ji} =$	$GV_{ji}c_j + \varepsilon_{ji}$	idem 1	idem 1	idem 1	idem 1
Number of runs	$R =$	100	idem 1	idem 1	idem 1	idem 1

* Correlations between all random quantities are zero unless otherwise specified

** Set of effective causal SNPs in study j, $\mathcal{M}_j \subset \mathcal{S}$, the set of effective SNPs

*** For each combination of values of h^2_{SNP}, fraction of overlapping causal SNPs, and CGR among overlapping SNPs, simulations are performed

**** The *Beta*(0.35,0.35) distribution fits the empirical distribution of allele frequencies in the Health and Retirement Study data well

***** '$=$' denotes $f_{jk} = f_{hk}$ and '$\perp\!\!\!\perp$' denotes f_{jk} is independent of f_{hk}

is carried out in each of the three discovery samples. GWAS results are then meta-analyzed using sample-size weighting. The fraction of causal SNPs reaching genome-wide significance in the meta-analysis is the estimate of statistical power per SNP. The squared correlation between the meta-analysis-based PGS for the hold-out sample and the corresponding phenotype is the estimate of the PGS R^2.

Final estimates of power per causal SNPs and PGS R^2 are obtained by averaging the estimates across the runs. Fig. A1–A2, show the resulting estimates of power per causal SNP in the meta-analysis and the R^2 of the PGS, for both Simulations 1–4 and Simulation 5. In addition, both figures report the power per causal SNP and R^2 predicted by the theoretical model, derived under the assumptions discussed in S1 Derivations. Inspection of Fig. A1 shows that there is no qualitative difference between the contour plots. Moreover, when computing the root-mean-square error (RMSE) between the theoretical predictions and the simulation-based estimates of power and R^2, even for the most extreme departures from our assumptions regarding allele frequencies and effects sizes (Simulations 3–4), the RMSE in power remains below 3% and the RMSE in R^2 of the PGS below 2%. Hence, the theoretical predictions of GWAS power and predictive accuracy – derived under assumptions of equal true R^2 of causal SNPs, with uniformly distributed allele frequencies that are equal across studies – are robust to violations of these assumptions.

Inspection of Fig. A2 shows that when CGRs are being shaped by a combination of poor overlap and poorly correlated effects of overlapping loci, there are some qualitative differences between predicted power and predictive accuracy compared to simulation-based estimates. However, the RMSE of theoretical power is only 1.2% with respect to the power estimated from simulations. Similarly, the RMSE of theoretical predictive accuracy is only 1.3%. Hence, the quantitative differences are small.

Simulation 5 shows that when low CGRs are induced by poor overlap of causal loci across studies instead of low correlations of the effects of overlapping loci, this leads to a slight downward bias in our theoretical predictions (i.e., making our theory conservative). Hence, we argue that if our calculator deems a study design well-powered, the analyses will be well powered, potentially even more so than what our theory predicts (e.g., if some of the imperfect CGR can be attributed to causal loci that are not shared across studies).

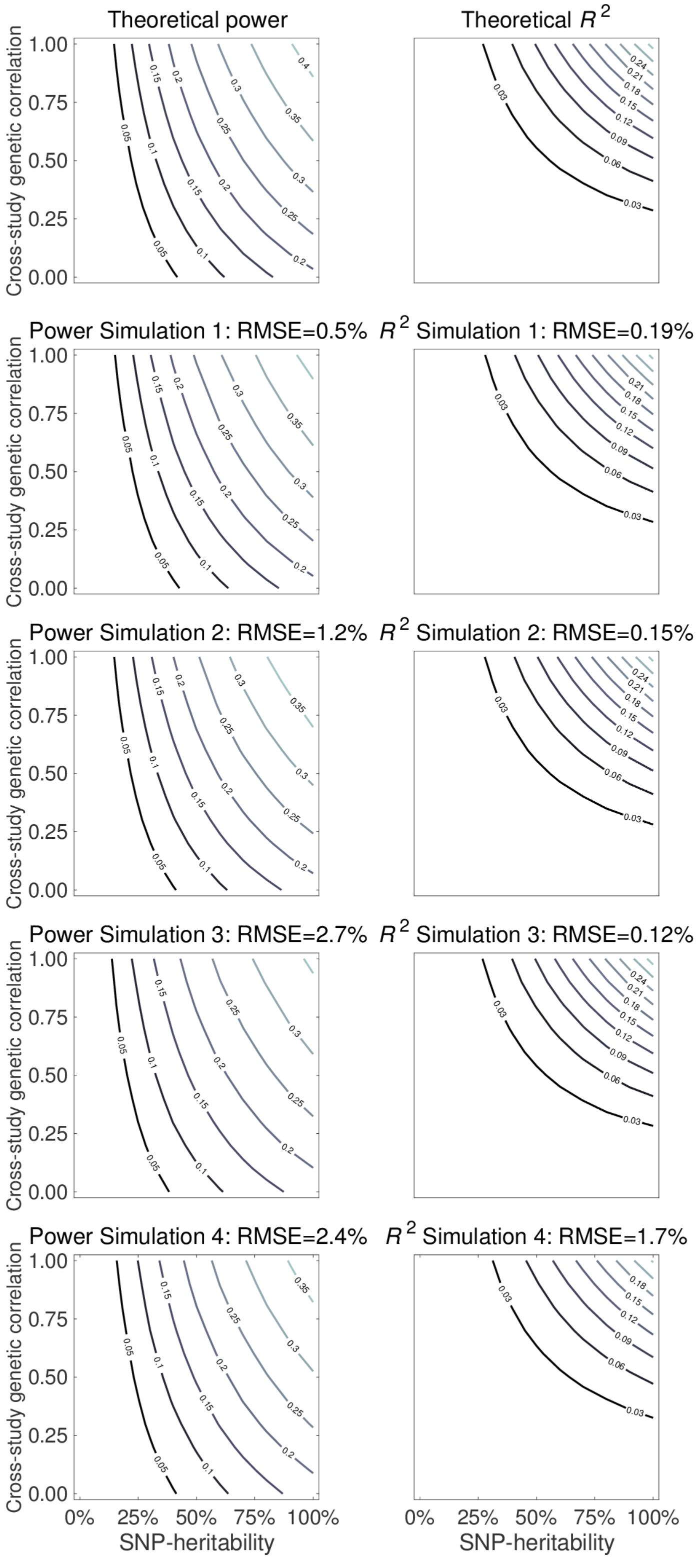

Figure A1. Power and polygenic score R^2 contour plots with, in each plot, SNP heritability on the x-axis and cross-study genetic correlation on the y-axis. The first row shows predictions from the theoretical model. Subsequent rows show estimates based on respective simulation studies. The first column shows power per causal SNP. The second column the R^2 of a polygenic score in a hold-out sample. Above each plot, the root-mean-square error (RMSE) is reported for the difference between predictions from the theoretical model and the simulation-based estimates.

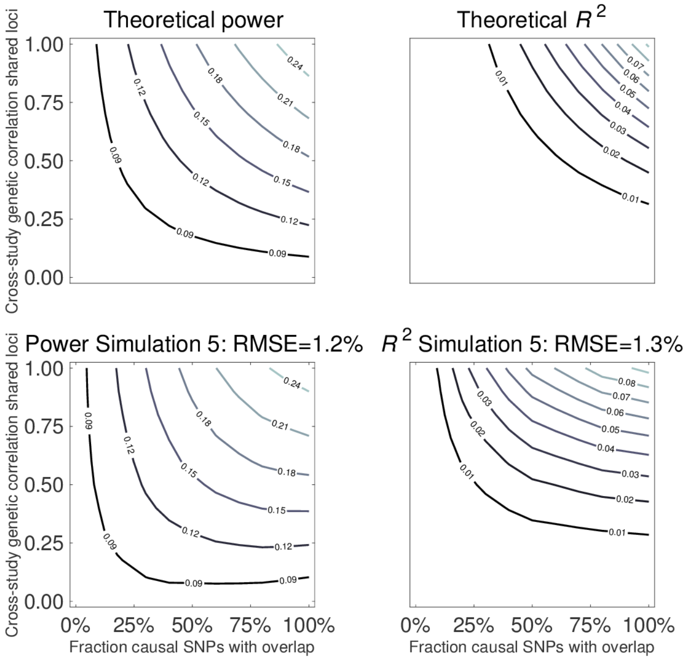

Figure A2. Power and polygenic score R^2 contour plots, with in each plot the fraction of causal loci that overlaps across studies on the x-axis and the cross-study correlation of the effects of overlapping loci on the y-axis. The first row shows predictions from the theoretical model. The second row shows estimates based on a simulation study. The first column shows power per causal SNP. The second column the R^2 of a polygenic score in a hold-out sample. Above each plot, the root-mean-square error (RMSE) is reported for the difference between predictions from the theoretical model and the simulation-based estimates.
