# Supplementary material for: Meta-GWAS Accuracy and Power (MetaGAP) Calculator Shows that Hiding Heritability Is Partially Due to Imperfect Genetic Correlations across Studies
Source: PLoS Genet. 2017 Jan 17;13(1):e1006495. doi: 10.1371/journal.pgen.1006495 (PMC5240919; doi:10.1371/journal.pgen.1006495)
Supplement: S1 Data — (PDF) [file pgen.1006495.s005.pdf]

S1 Data. Description of genotype and phenotype data, and quality control.

Genotype data In the bivariate and univariate genomic-relatedness-matrix restricted maximum likelihood (GREML) analyses we use genotype data from the Rotterdam Study (RS; Ergo waves 1-4 sample denoted by RS-I, Ergo Plus sample denoted by RS-II, and Ergo Jong sample denoted by RS-III), the Swedish Twin Registry (STR; TwinGene sample), and the Health and Retirement Study (HRS). For each study, details on the genotyping platform, quality control (QC) prior to imputation, the reference sample used for imputation, and imputation software, are listed in Table A1.

Table A1. Genotyping and imputation

Study	Genotyping platform	SNP exclusions			Subject exclusions*	Imputation**
		MAF <	Call rate <	HWE p-val. <	Call rate <	Software
RS-I	Illumina 550K	0%	97.5%	10^{-7}	97.5%	MaCH/Minimac
RS-II	Illumina 550K	0%	97.5%	10^{-7}	97.5%	MaCH/Minimac
RS-III	Illumina 610K	0%	97.5%	10^{-7}	97.5%	MaCH/Minimac
STR	HumanOmniExpress 12v1A	1%	97.0%	10^{-7}	97.0%	MaCH/Minimac
HRS	Illumina Omni2.5	1%	98.0%	10^{-4}	98.0%	IMPUTE2

* Individuals are also excluded on the basis of sex mismatch, close relatives, duplicates and ancestry outliers (STR excepted), or autosomal heterozygosity outliers (HRS excepted)
** All samples have been imputed against the 1000 Genomes, Phase 1, Version 3 haplotypes of all ancestries.

To increase the overlap of SNPs across studies, we use genotypes imputed on the basis of the 1000 Genomes, Phase 1, Version 3 reference panel [1]. We only consider the subset of HapMap3 SNPs [2] available in the 1000-Genomes data. By using this subset we substantially reduce the computational burden of the analyses, while preserving overlap between the SNP-sets in the studies and still having a sufficiently dense set of both common and more rare SNPs (# SNPs after QC $\approx$ 1 million).

Quality control Prior to QC, we extract only SNPs that are in the HapMap3 reference sample (source: `http://hapmap.ncbi.nlm.nih.gov/downloads/genotypes/hapmap3_r3/plink_format/`, accessed: December 11, 2014) from the imputed genotype data of each study and convert the allele dosages to best-guess `PLINK` [3, 4] binary files by rounding dosages using `GCTA` [5]. Subsequently, we perform QC on the best-guess genotypes in two stages. In the first stage, we clean and harmonize the imputed genotype data at the study level. The cleaned and harmonized study genotypes are then merged into a pooled dataset. The second round of QC is aimed at cleaning the pooled dataset, on the basis of the samples for which the phenotype is available. Hence, the first QC stage is phenotype-independent, whereas the second stage depends on the phenotype of interest.

In the first QC stage (prior to merging), we filter out the following markers and individuals:

1. SNPs with imputation accuracy below 70%.

2. Non-autosomal SNPs.

3. SNPs with minor allele frequency below 1%.

4. SNPs with Hardy-Weinberg-Equilibrium-test p-value below 1%.

5. SNPs with missingness (i.e., fraction of data that is missing) greater than 5%.

6. Individuals with missingness greater than 5%.

7. SNPs that are not present in all studies.

8. SNPs of which the alleles cannot be aligned across studies.

Prior to the first QC stage, we apply the following two additional steps in HRS:

1. Switch alleles to address a strand-flip error due to incorrect annotation.

2. Drop individuals of non-European ancestry.

After the first round of QC, a set of roughly 1 million overlapping SNPs, available for about 30,000 individuals is left. Panel I in Table A2 shows, for each study, the number of SNPs and individuals before and after the first round of QC.

The second QC stage, applied to the pooled data set, comprises the following steps:

1. Keep only individuals for whom the phenotype of interest and all corresponding control variables are available.

2. Drop SNPs with a minor allele frequency below 1%.

3. Drop SNPs with Hardy-Weinberg-Equilibrium p-value below 1%.

4. Drop SNPs with missingness greater than 5%.

5. Drop individuals with missingness greater than 5%.

6. Keep only one individual per pair of individuals with a genomic relatedness greater than 0.025.

Since the data in STR consists of twins and having highly related individuals can bias estimates of SNP-based heritability due to environment-sharing, we randomly select only one individual per twin pair after Step 1 in the second QC stage.

Panel II in Table A2 shows the sample size and the number of SNPs in the pooled dataset for the phenotypes discussed in the next subsection. We only consider phenotypes that attain a sample size of at least 18,000 individuals after all QC steps. For all phenotypes, the number of SNPs is slightly greater than one million.

Table A2. Number of individuals and SNPs before and after quality control (QC) at the study level (Panel I) and at the pooled level (Panel II).

**Panel I: study-level QC**				
Study	*N*		# SNPs	
	pre-QC	post-QC	pre-QC	post-QC
RS-I	6,291	6,291	31,337,615	1,062,589
RS-II	2,157	2,157	31,337,615	1,062,589
RS-III	3,048	3,048	31,337,615	1,062,589
STR	9,617	9,617	31,326,389	1,062,589
HRS	12,454	8,652	21,632,048	1,062,589
Total		29,765		1,062,589
**Panel II: pooled-level QC**				
Phenotype	*N*		# SNPs	
	pre-QC	post-QC	pre-QC	post-QC
Height	29,765	20,458	1,062,589	1,052,572
BMI	29,765	20,449	1,062,589	1,052,600
EduYears	29,765	20,619	1,062,589	1,052,626
CurrCigt	29,765	20,686	1,062,589	1,052,524
CurrDrinkFreq	29,765	20,072	1,062,589	1,052,958
Self-rated health	29,765	19,184	1,062,589	1,053,190

Phenotype data For HRS, we use the RAND HRS data, version N, to obtain the phenotypes of interest. These data consist of measurements from eleven waves. RS-I consists of four data waves (Ergo 1-4). In both HRS and RS-I, data for some phenotypes are only available in a subset of the waves. RS-II, RS-III and STR do not have multiple measures over time for the phenotypes considered in this study. Table A3 describes how the phenotypes are constructed in each of the five studies.

As Table A3 shows, height, BMI, *EduYears*, and *CurrCigt* are measured quite consistently across waves. The self-rated health phenotype is also measured quite consistently, although in RS respondents are asked about health compared to members of the same age group, whereas a more absolute question is posed in STR and HRS. The drinking measure *CurrFreqDrink* is also measured somewhat heterogeneously; the threshold for what we treat as 'frequent drinking' is determined by how fine-grained the drinking frequency measure is in the respective studies.

Table A3. Study-level phenotype measures.

Phenotype	Survey instrument in				
	RS-I	RS-II	RS-III	STR	HRS
Years of education (*EduYears*)	Constructed in line with [6] in all studies.				
Height	Median height across waves 1-4.	Height	Height	Height	Median height across waves 1-11
BMI	Median BMI across waves 1-4.	BMI	BMI	BMI	Median BMI across waves 1-11
Currently smoking cigarettes (*CurrCigt*)	1 if stated to be a current smoker of cigarettes in the latest available measurement across waves 1-4.	1 if stated to be a current cigarette smoker.	Same as RS-II.	1 if stated to be a current cigarette smoker.	1 if responded positively to “currently smokes cigarettes?” in the latest available measurement across waves 1-11.
Currently drinking frequently (*CurrDrinkFreq*)	1 if indicated to “drink one or more alcoholic beverages per week” in the latest available measurement across waves 1-4.	1 if indicated to “drink one or more alcoholic beverages per week”.	1 if indicated to “have drunk at least two alcoholic beverages a month during the the past year.”	1 if indicated to “have drunk at least two alcoholic beverages in the past month”.	1 if indicated to “drink alcohol once per week or more” in the latest available measurement across waves 3-11.
Self-rated health	Only available in wave 1: “How is your general health compared to members of your age group?” Response categories reverse-coded such that 0=worse, 1=same, and 2=better.	Same as RS-I.	*n.a.*	Rate their general health. Response categories re-coded such that 0=bad, 1=not so good, 2=average, 3=good, 4=excellent.	Mode of the 4-point self-reported health measure in HRS across waves 1-11. Responses reverse-coded such that 0=poor, 1=fair, 2=good, 3=very good, and 4=excellent.

References

1. McVean GA, Altshuler DM, the 1000 Genomes Project Consortium. An integrated map of genetic variation from 1,092 human genomes. Nature. 2012;491:56–65.

2. Altshuler DM, Gibbs RA, the International HapMap 3 Consortium. Integrating common and rare genetic variation in diverse human populations. Nature. 2010;467:52–58.

3. Purcell SM, Neale B, Todd-Brown K, Thomas L, Ferreira MAR, Bender D, et al. PLINK: a tool set for whole-genome association and population-based linkage analyses. Am J Hum Genet. 2007;81:559–575.

4. Chang CC, Chow CC, Tellier LCAM, Vattikuti S, Purcell SM, Lee JJ. Second-generation PLINK: rising to the challenge of larger and richer datasets. GigaScience. 2015;4:1–16.

5. Yang J, Lee SH, Goddard ME, Visscher PM. GCTA: a tool for genome-wide complex trait analysis. Am J Hum Genet. 2011;88:76–82.

6. Rietveld CA, Medland SE, Derringer J, Yang J, Esko T, Martin NW, et al. GWAS of 126,559 individuals identifies genetic variants associated with educational attainment. Science. 2013;340:1467–1471.
