# Supplementary material for: Meta-GWAS Accuracy and Power (MetaGAP) Calculator Shows that Hiding Heritability Is Partially Due to Imperfect Genetic Correlations across Studies
Source: PLoS Genet. 2017 Jan 17;13(1):e1006495. doi: 10.1371/journal.pgen.1006495 (PMC5240919; doi:10.1371/journal.pgen.1006495)
Supplement: S1 Estimation — (PDF) [file pgen.1006495.s006.pdf]

S1 Estimation. GREML estimation procedure.

Height, BMI, *EduYears*, and self-rated health are treated as quantitative traits. *CurrCigt* and *CurrDrinkFreq* are treated as binary outcomes. In each study, (after aggregating across waves, if applicable) we regress quantitative phenotypes on age, squared age, sex, and an intercept. The residuals from the regression are standardized to have a sample-mean equal to zero and variance equal to one. For both binary and quantitative traits, the aforementioned covariates are also included in the GREML estimation. In addition, in bivariate GREML and pooled GREML estimation (i.e., considering multiple studies jointly), the intercept is replaced by indicator variables for the respective studies, capturing study-specific fixed effects. Finally, 20 principal components from the phenotype-specific genomic-relatedness matrix are added to the set of control variables in the GREML estimation, in order to correct for population stratification [1].

References

1. Price AL, Patterson NJ, Plenge RM, Weinblatt ME, Shadick NA, Reich D. Principal components analysis corrects for stratification in genome-wide association studies. Nat Genet. 2006;38:904–909.
