# Supplementary material for: Meta-GWAS Accuracy and Power (MetaGAP) Calculator Shows that Hiding Heritability Is Partially Due to Imperfect Genetic Correlations across Studies
Source: PLoS Genet. 2017 Jan 17;13(1):e1006495. doi: 10.1371/journal.pgen.1006495 (PMC5240919; doi:10.1371/journal.pgen.1006495)
Supplement: S1 Table — (PDF) [file pgen.1006495.s007.pdf]

S1 Table. GREML estimates of SNP heritability and genetic correlation across studies.

Phenotype	*N*				Univariate estimates SNP heritability[1]				Bivariate estimates genetic correlation[1,2]			
	RS	STR	HRS	Total	RS	STR	HRS	Average[3]	RS–STR	RS–HRS	STR–HRS	Average[4]
Height	6,780	5,342	8,336	20,458	48.9% (4.9%) ***	50.8% (6.0%) ***	37.9% (4.1%) ***	44.9%	0.976 (0.102) ***	0.954 (0.095) ***	0.967 (0.106) ***	0.965
BMI	6,775	5,341	8,333	20,449	28.9% (4.9%) ***	16.4% (6.1%) ***	19.6% (4.1%) ***	21.9%	1.000 (0.269) ***	0.914 (0.172) ***	0.847 (0.246) ***	0.917
EduYears	6,735	5,543	8,341	20,619	17.5% (4.8%) ***	20.6% (5.8%) ***	17.3% (4.0%) ***	18.2%	0.690 (0.233) ***	0.659 (0.224) ***†	1.000 (0.263) ***	0.783
CurrCigt	6,803	5,579	8,304	20,686	17.8% (10.1%) **	18.7% (13.8%) *	20.4% (11.2%) **	19.1%	1.000 (0.643) ***	0.611 (0.448) *	1.000 (0.607) ***	0.858
CurrDrinkFreq	6,172	5,564	8,336	20,072	13.5% (8.7%) *	14.1% (9.5%) *	5.3% (6.3%)	10.3%	1.000 (0.666) ***	0.298 (0.670)	-0.056 (0.647)	0.381
Self-rated health	5,264	5,577	8,343	19,184	13.5% (6.2%) **	9.4% (5.7%) **	21.3% (4.0%) ***	15.7%	0.626 (0.439) **	0.363 (0.223) **††	0.447 (0.278) **	0.468

[1] Standard errors between parentheses.
[2] Significance of deviations from one only tested for genetic correlations.
[3] Sample-size weighted averages of univariate estimates across studies.
[4] Sample-size weighted averages of bivariate estimates across pairs of studies.

* > 0 at 10% sign. † < 1 at 10% sign.
** > 0 at 5% sign. †† < 1 at 5% sign.
*** > 0 at 1% sign. ††† < 1 at 1% sign.
