# Supplementary material for: Meta-GWAS Accuracy and Power (MetaGAP) Calculator Shows that Hiding Heritability Is Partially Due to Imperfect Genetic Correlations across Studies
Source: PLoS Genet. 2017 Jan 17;13(1):e1006495. doi: 10.1371/journal.pgen.1006495 (PMC5240919; doi:10.1371/journal.pgen.1006495)
Supplement: S2 Table — (PDF) [file pgen.1006495.s008.pdf]

S2 Table. GREML estimates of SNP heritability and genetic correlation across sexes.

**Phenotype**	***N***			**Univariate estimates SNP heritability**[1]			**Bivariate estimates genetic correlation**[1,2]
	**Females**	**Males**	**Total**	**Females**	**Males**	**Average**[3]	**Females–Males**
Height	11,553	8,905	20,458	43.2% (3.0%) ***	45.1% (3.8%) ***	44.0%	0.981 (0.067) ***
BMI	11,542	8,907	20,449	22.1% (2.9%) ***	23.8% (3.8%) ***	22.8%	0.794 (0.122) *** †
EduYears	11,653	8,966	20,619	18.1% (2.9%) ***	18.9% (3.7%) ***	18.4%	0.832 (0.162) ***
CurrCigt	11,706	8,980	20,686	22.3% (7.1%) ***	26.7% (9.1%) ***	24.2%	0.543 (0.257) *** †
CurrDrinkFreq	11,312	8,760	20,072	14.1% (4.6%) ***	0.9% (6.0%)	8.3%	1.000 (2.068) *
Self-rated health	10,866	8,318	19,184	8.6% (3.1%) ***	10.8% (4.0%) ***	9.5%	1.000 (0.349) ***

[1] Standard errors between parentheses.
[2] Significance of deviations from one only tested for genetic correlations.
[3] Sample-size weighted averages of univariate estimates in females and males.

* > 0 at 10% sign. † < 1 at 10% sign.
** > 0 at 5% sign. †† < 1 at 5% sign.
*** > 0 at 1% sign. ††† < 1 at 1% sign.
