# Supplementary material for: Meta-GWAS Accuracy and Power (MetaGAP) Calculator Shows that Hiding Heritability Is Partially Due to Imperfect Genetic Correlations across Studies
Source: PLoS Genet. 2017 Jan 17;13(1):e1006495. doi: 10.1371/journal.pgen.1006495 (PMC5240919; doi:10.1371/journal.pgen.1006495)
Supplement: S3 Table — (PDF) [file pgen.1006495.s009.pdf]

S3 Table. Details and notes on the sources for the sample size, the number of meta-analyzed studies, the number of genome-wide-significant hits, and the PGS R^2, for large-scale GWAS efforts to date for height, BMI, *EduYears*, and self-rated health, reported in the same order as in Table 2.

Study	*N*			*C**			Number of hits			PGS R^2		
	Section	Page	Note	Section	Page	Note	Section	Page	Note	Section	Page	Note
Wood *et al.* (2014) [1]	Abstract	1173		Results	1173		Abstract	1173		Figure 2d	1175	Approximation least stringent p-value threshold
Allen *et al.* (2010) [2]	Abstract	832		SI	2	Stages 1–2	Abstract	832		Figure 1a	833	Approximation least stringent p-value threshold
Weedon *et al.* (2008) [3]	Abstract	575		Results	576		Table 1	577		Results	580	PGS based on 20 SNPs
Locke *et al.* (2015) [4]	Abstract	197		Results	197		Abstract	197		Extended Data Figure 3c		Approximation
Speliotes *et al.* (2010) [5]	Abstract	937		Results	937		Results	937		Results	941	Least stringent p-value threshold
Willer *et al.* (2008) [6]	Results	26	Stage 1	Results	26	Stage 1	Results	26	Stage 1	*n.a.*		
Okbay *et al.* (2016) [7]	SI	16		SI	12,16		SI	16		*n.a.*		
Okbay *et al.* (2016) [7]	Abstract	539		SI	12		Abstract	539		SI	108	
Rietveld *et al.* (2013) [8]	Abstract	1467		Results	1467		Results	1467	EduYears hits	Results	1469	Approximation average
Harris *et al.* (2016) [9]	Abstract	1		Abstract	1		Abstract	1		*n.a.*		

*C denotes the number of studies in the meta-analysis; C is slightly subjective (e.g., RS I, II, and III can be considered as one study or as three).

References

1. Wood AR, Esko T, Yang J, Vedantam S, Pers TH, Gustafsson S, et al. Defining the role of common variation in the genomic and biological architecture of adult human height. Nat Genet. 2014;46:1173–1186.

2. Lango Allen H, Estrada K, Lettre G, Berndt SI, Weedon MN, Rivadeneira F, et al. Hundreds of variants clustered in genomic loci and biological pathways affect human height. Nature. 2010;467:832–838.

3. Weedon MN, Lango Allen H, Lindgren CM, Wallace C, Evans DM, Mangino M, et al. Genome-wide association analysis identifies 20 loci that influence adult height. Nat Genet. 2008;40:575–583.

4. Locke AE, Kahali B, Berndt SI, Justice AE, Pers TH, Day FR, et al. Genetic studies of body mass index yield new insights for obesity biology. Nature. 2015;518:197–206.

5. Speliotes EK, Willer CJ, Berndt SI, Monda KL, Thorleifsson G, Jackson AU, et al. Association analyses of 249,796 individuals reveal 18 new loci associated with body mass index. Nat Genet. 2010;42:937–948.

6. Willer CJ, Speliotes EK, Loos RJF, Li S, Lindgren CM, Heid IM, et al. Six new loci associated with body mass index highlight a neuronal influence on body weight regulation. Nat Genet. 2008;41:25–34.

7. Okbay A, Beauchamp JP, Fontana MA, Lee JJ, Pers TH, Rietveld CA, et al. Genome-wide association study identifies 74 loci associated with educational attainment. Nature. 2016;533:539–542.

8. Rietveld CA, Medland SE, Derringer J, Yang J, Esko T, Martin NW, et al. GWAS of 126,559 individuals identifies genetic variants associated with educational attainment. Science. 2013;340:1467–1471.

9. Harris SE, Hagenaars SP, Davies G, Hill WD, Liewald DCM, Ritchie SJ, et al. Molecular genetic contributions to self-rated health. Int J Epidemiol. 2016;advance access:dyw219.
