# Supplementary material for: Meta-GWAS Accuracy and Power (MetaGAP) Calculator Shows that Hiding Heritability Is Partially Due to Imperfect Genetic Correlations across Studies
Source: PLoS Genet. 2017 Jan 17;13(1):e1006495. doi: 10.1371/journal.pgen.1006495 (PMC5240919; doi:10.1371/journal.pgen.1006495)
Supplement: S4 Table — (PDF) [file pgen.1006495.s010.pdf]

S4 Table. Meta-analysis methods used in large-scale GWAS efforts to date for height, BMI, *EduYears*, and self-rated health, reported in the same order as in Table 2.

**Phenotype**	**Large-scale GWAS**	**Meta-analysis**			
		**Software**	**Weighting scheme**	**Effects**	**Accounts for heterogeneity**
Height	Wood *et al.* (2014) [1]	`METAL`	Inverse-variance	Fixed	No
	Allen *et al.* (2010) [2]	`METAL`	Inverse-variance	Fixed	No
	Weedon *et al.* (2008) [3]*	*n.a.*	Inverse-variance	Fixed	No
BMI	Locke *et al.* (2015) [4]	`METAL`	Inverse-variance	Fixed	No
	Speliotes *et al.* (2010) [5]	`METAL`	Inverse-variance and sample-size	Fixed	No
	Willer *et al.* (2008) [6]	`METAL`	Sample-size	Fixed	No
EduYears	Okbay *et al.* (2016) [7]	`METAL`	Sample-size	Fixed	No
	Okbay *et al.* (2016) [7]	`METAL`	Sample-size	Fixed	No
	Rietveld *et al.* (2013) [8]	`METAL`	Sample-size	Fixed	No
Self-rated health	Harris *et al.* (2016) [9]**	*n.a.*	*n.a.*	*n.a.*	*n.a.*

* No commonly-used meta-analysis tool is applied.
** No meta-analysis is used since this concerns data from a single study.

References

1. Wood AR, Esko T, Yang J, Vedantam S, Pers TH, Gustafsson S, et al. Defining the role of common variation in the genomic and biological architecture of adult human height. Nat Genet. 2014;46:1173–1186.

2. Lango Allen H, Estrada K, Lettre G, Berndt SI, Weedon MN, Rivadeneira F, et al. Hundreds of variants clustered in genomic loci and biological pathways affect human height. Nature. 2010;467:832–838.

3. Weedon MN, Lango Allen H, Lindgren CM, Wallace C, Evans DM, Mangino M, et al. Genome-wide association analysis identifies 20 loci that influence adult height. Nat Genet. 2008;40:575–583.

4. Locke AE, Kahali B, Berndt SI, Justice AE, Pers TH, Day FR, et al. Genetic studies of body mass index yield new insights for obesity biology. Nature. 2015;518:197–206.

5. Speliotes EK, Willer CJ, Berndt SI, Monda KL, Thorleifsson G, Jackson AU, et al. Association analyses of 249,796 individuals reveal 18 new loci associated with body mass index. Nat Genet. 2010;42:937–948.

6. Willer CJ, Speliotes EK, Loos RJF, Li S, Lindgren CM, Heid IM, et al. Six new loci associated with body mass index highlight a neuronal influence on body weight regulation. Nat Genet. 2008;41:25–34.

7. Okbay A, Beauchamp JP, Fontana MA, Lee JJ, Pers TH, Rietveld CA, et al. Genome-wide association study identifies 74 loci associated with educational attainment. Nature. 2016;533:539–542.

8. Rietveld CA, Medland SE, Derringer J, Yang J, Esko T, Martin NW, et al. GWAS of 126,559 individuals identifies genetic variants associated with educational attainment. Science. 2013;340:1467–1471.

9. Harris SE, Hagenaars SP, Davies G, Hill WD, Liewald DCM, Ritchie SJ, et al. Molecular genetic contributions to self-rated health. Int J Epidemiol. 2016;advance access:dyw219.
